# Novel nanofibrous membrane‐supporting stem cell sheets for plasmid delivery and cell activation to accelerate wound healing

**DOI:** 10.1002/btm2.10244

**Published:** 2021-08-12

**Authors:** Yanxia Zhu, Yuqi Liao, Yuanyuan Zhang, Mehdihasan I. Shekh, Jianhao Zhang, Ziyang You, Bing Du, Cuihong Lian, Qianjun He

**Affiliations:** ^1^ Shenzhen Key Laboratory for Anti‐ageing and Regenerative Medicine, Department of Medical Cell Biology & Genetics, Health Science Center Shenzhen University Shenzhen China; ^2^ Guangdong Provincial Key Laboratory of Biomedical Measurements and Ultrasound Imaging, National‐Regional Key Technology Engineering Laboratory for Medical Ultrasound, School of Biomedical Engineering, Health Science Center Shenzhen University Shenzhen China; ^3^ Department of Dermatology The First Affiliated Hospital of Shenzhen University Shenzhen China; ^4^ College of Materials Science and Engineering, Shenzhen Key Laboratory of Polymer Science and Technology, Guangdong Research Center for Interfacial Engineering of Functional Materials, Nanshan District Key Lab for Biopolymers and Safety Evaluation Shenzhen University Shenzhen China

**Keywords:** cell sheet, chitosan, gelatin, stem cell, sustained release, tissue engineering, wound healing

## Abstract

The integration of biomaterials with cells for high overall performances is vitally important in tissue engineering, as scaffold‐free cell sheet lacks enough mechanical performance and cell viability while cell‐free scaffold possesses limited biological functions. In this study, we propose a new strategy to strengthen cell sheets and enhance cell activity for accelerating wound healing based on a novel sandwich structure of cell sheet‐plasmid@membrane‐cell sheet (CpMC). Specifically, the CpMC contains two adipose‐derived stem cell (ADSC) sheets on outer surfaces and an electrospun gelatin/chitosan nanofibrous membrane (NFM) encapsulating vascular endothelial growth factor (VEGF) plasmids in between. The physicochemical properties of NFM including swelling, stiffness, strength, elasticity, and biodegradation can be tailored by simply adjusting the ratio between gelatin and chitosan to be 7:3 which is optimal for most effectively supporting ADSCs adhesion and proliferation. The swelling/biodegradation of NFM mediates the sustained release of encapsulated VEGF plasmids into adjacent ADSCs, and NFM assists VEGF plasmids to promote the differentiation of ADSCs into endothelial, epidermal, and fibroblast cells, in support of the neoangiogenesis and regeneration of cutaneous tissues within 2 weeks. The proposed membrane‐supporting cell sheet strategy provides a new route to tissue engineering, and the developed CpMC demonstrates a high potential for clinical translation.

## 1 INTRODUCTION

Regenerative medicine is promoted as a promising treatment for the difficult‐to‐treat diseases with physically impaired function in patients.^1^ Replacement of injured or lost tissues with appropriate cells or tissues is one of most desired treatments in regenerative medicine. Tissue engineering is a promising strategy to overcome inadequate cell number and cell loss after transplantation.^1^ The integration of scaffold with functional cells is an important route to enhance regeneration efficacy, but their biocompatibility and cellular activity maintenance are still challenging.^2^ On the other hand, Okano et al. proposed the concept of scaffold‐free cell sheet engineering to bypass the effect of scaffold.^3^ But it is challenging to facilely harvest intact cell sheet because of low strength and poor stability of extracellular matrix (ECM). Moreover, the layering of multiple cell sheets severely confines cell growth and nutrient supply, impairing cell viability and differentiation potential. Therefore, we here proposed a new strategy to strengthen cell sheets and activate cells based on porous membrane supporting cell sheets for the first time.

Since collagen is the primary constituent of ECM,^4^ gelatin is a denatured form of collagen and therefore is being popularly used in tissue engineering owing to its high biodegradation, bioresolvability, biocompatibility, commercial availability, and nonantigenicity.^5^ Chitosan is also a kind of promising biopolymers for tissue engineering because of its biocompatibility, biodegradability, and antibacterial and antifungal activity.^6^ The electrospinning technique is a simple and versatile method to fabricate nanofibrous membrane (NFM), whose morphology can closely mimic the ECM structure and thus facilitate cell adhesion, proliferation, and differentiation.^7^, ^8^, ^9^, ^10^, ^11^ The use of electrospinning technique to make NFM with cross‐linked gelatin/chitosan mixture can well overcome the shortcomings of overquick biodegradation of gelatin and low spinnability of chitosan,^12^ but the effect of the gelatin/chitosan proportion on the physicochemical and biochemical properties of gelatin/chitosan NFM is unknown so far.

**SCHEME 1:**
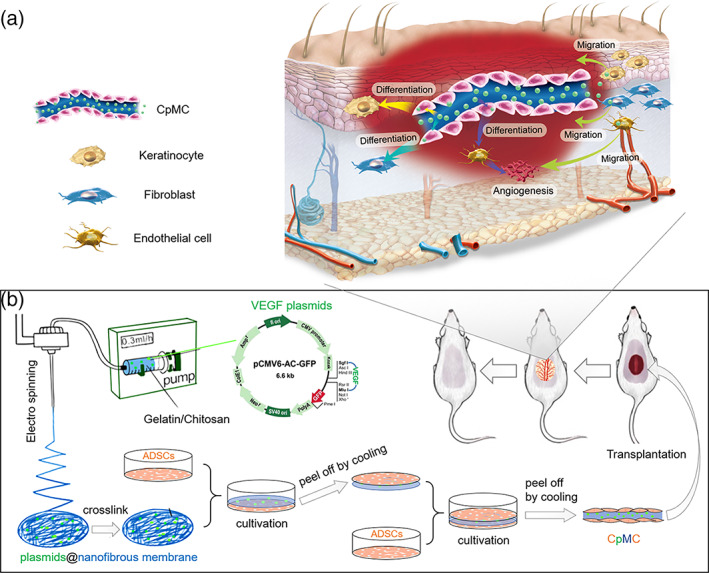
Schematic illustration of the fabrication of the sandwich structure of cell sheet‐plasmid@membrane‐cell sheet (CpMC) with electrospun gelatin/chitosan nanofibrous membrane (NFM) and two‐layer adipose‐derived stem cell (ADSC) sheets for wound healing (b), and the mechanisms for skin regeneration (a)

In this work, we synthesized a biodegradable gelatin/chitosan NFM by an electrospinning method, and used it to construct a novel sandwich structure by supporting two adipose‐derived stem cell (ADSC) sheets on both sides (cell sheet‐membrane‐cell sheet, abbreviated as CMC) for efficiently repairing wound (Scheme 1). High porosity, hydrophilicity, and mechanical properties of the NFM with an optimal proportion of gelatin/chitosan (7:3) effectively strengthened cell sheets and activated supported ADSCs, including the enhancement of cellular activity and the facilitation of ADSCs differentiation into various skin cells. Moreover, the electrospinning technique enabled local encapsulation of vascular endothelial growth factor (VEGF) plasmids into the nanofibers (cell sheet‐plasmid@membrane‐cell sheet, abbreviated as CpMC), whose excellent swelling/biodegradation features ensured sustained release of VEGF plasmids, which promoted the proliferation of various skin cells and their migration from normal tissue to wound site, and also enhanced the differentiation of endothelial cells to promote neoangiogenesis, in great support of wound healing and skin regeneration.

## 2 RESULTS

### 2.1 Construction and physicochemical characterization of NFM

**FIGURE 1:**
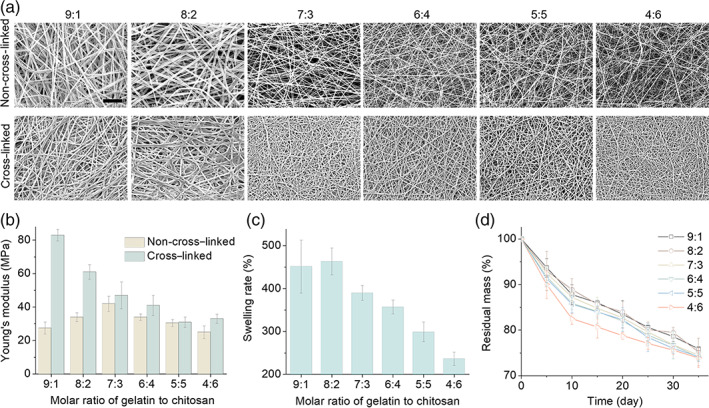
Characterization of electrospun nanofibrous membrane (NFM) with different gelatin/chitosan proportions. (a) Microstructure of different gelatin/chitosan NFM observed by scanning electron microscope (SEM). Scale bars: 20 μm. (b) Young's modulus of different gelatin/chitosan NFM before and after cross‐linking. (c) Water absorption ability of all the gelatin/chitosan NFM. (d) Degradation properties of all the gelatin/chitosan NFM

A series of the NFMs with various gelatin/chitosan proportions (9:1–4:6) were developed by the electrospinning method, and their physicochemical performances were investigated. Pure gelatin or chitosan hardly forms the nanofibers by the electrospinning method owing to poor intermolecular attraction of gelatin and poor solubility of chitosan in the organic solvents. The mixture of gelatin and chitosan can form electrostatic and hydrogen bonding attractions between them, and thus enables the facile fabrication of NFM by the electrospinning method. From scanning electron microscope (SEM) images in Figure 1a, all the as‐prepared NFM possessed a nanofibers‐fabricated porous network structure, and the covalent cross‐linking (supported by Furrier transform‐infrared spectroscopy [FTIR] and DSC data, Figures S1 and S2 and Table S3) caused the interconnection of nanofibers to enhance the strength of the NFMs (Figure 1b) but well maintained the nanofibrous diameter (Figure S1) and porous network of the NFM (Figure 1a). Higher proportion of chitosan resulted in finer gelatin/chitosan nanofibers of the cross‐linked NFM (Figure 1a; Figure S3), possibly owing to improved flexibility (Figure 1b). Nevertheless, the strength of NFMs (32–82 MPa; Figure 1a) was remarkably higher than that of ECM, and thus can meet the need of facilely maintaining the integrity of cell sheets during transfer of cells‐adhered membrane.

The water swelling and biodegradation capability of NFM is highly desired to keep the humidity in the wound site and to enable degradation‐induced sustained drug release in favor of wound healing. Though the gelatin/chitosan NFMs can be fabricated by the electrospinning method, the non‐cross‐linked NFMs will quickly degrade/resolve in the phosphate‐buffered solution (PBS) within half an hour and therefore cannot be applied for tissue engineering. By comparison, the covalent cross‐linking can remarkably improve the stability of the gelatin/chitosan NFMs (Figure 1c,d). All the following investigation was based on the cross‐linked gelatin/chitosan NFMs without special note. From Figure 1c, the lowest gelatin/chitosan proportion of 9:1 exhibited the highest swelling degree of 450%, in correspond to highest strength (Figure 1b), possibly owing to least cross‐linking/nanofibers‐interconnected points (Figure 1a). Anyway, the NFM with the investigated lowest gelatin/chitosan proportion of 4:6 still exhibited a considerable swelling rate of 230%. Take the moderate gelatin/chitosan proportion of 7:3 as an example, about 400% swelling will expand pore size of the NFM to about 36 μm within 24 h (Figure S3) in great favor of cell spreading inside the NFM for ideal loading of cell sheets.

As to biodegradation, much different from the as‐synthesized non‐cross‐linked NFMs, the cross‐linked NFMs exhibited a sustained biodegradation behavior in the PBS (Figure 1d) whose pH value almost always remained to be 7.0 ± 0.1 within 35 days (Figure S2). The biodegradation rate of the NFM with lower gelatin/chitosan proportion was slightly faster (Figure 1d) although its water swelling rate was lower (Figure 1c), possibly owing to higher specific surface area (Figure 1a). Anyway, these NFMs exhibited a relatively uniform biodegradation rate during 35‐day immersion, and all of them achieved about 25% of biodegradation rate after immersion in the PBS for 35 days (Figure 1d), in favor of sustained drug release. For summary, the nanofiber diameter, pore size, swelling property, degradation property, Young's modulus, and pH value of NFMs were listed in Table S2.

### 2.2 ADSCs adhesion, spreading, and proliferation on NFM

**FIGURE 2:**
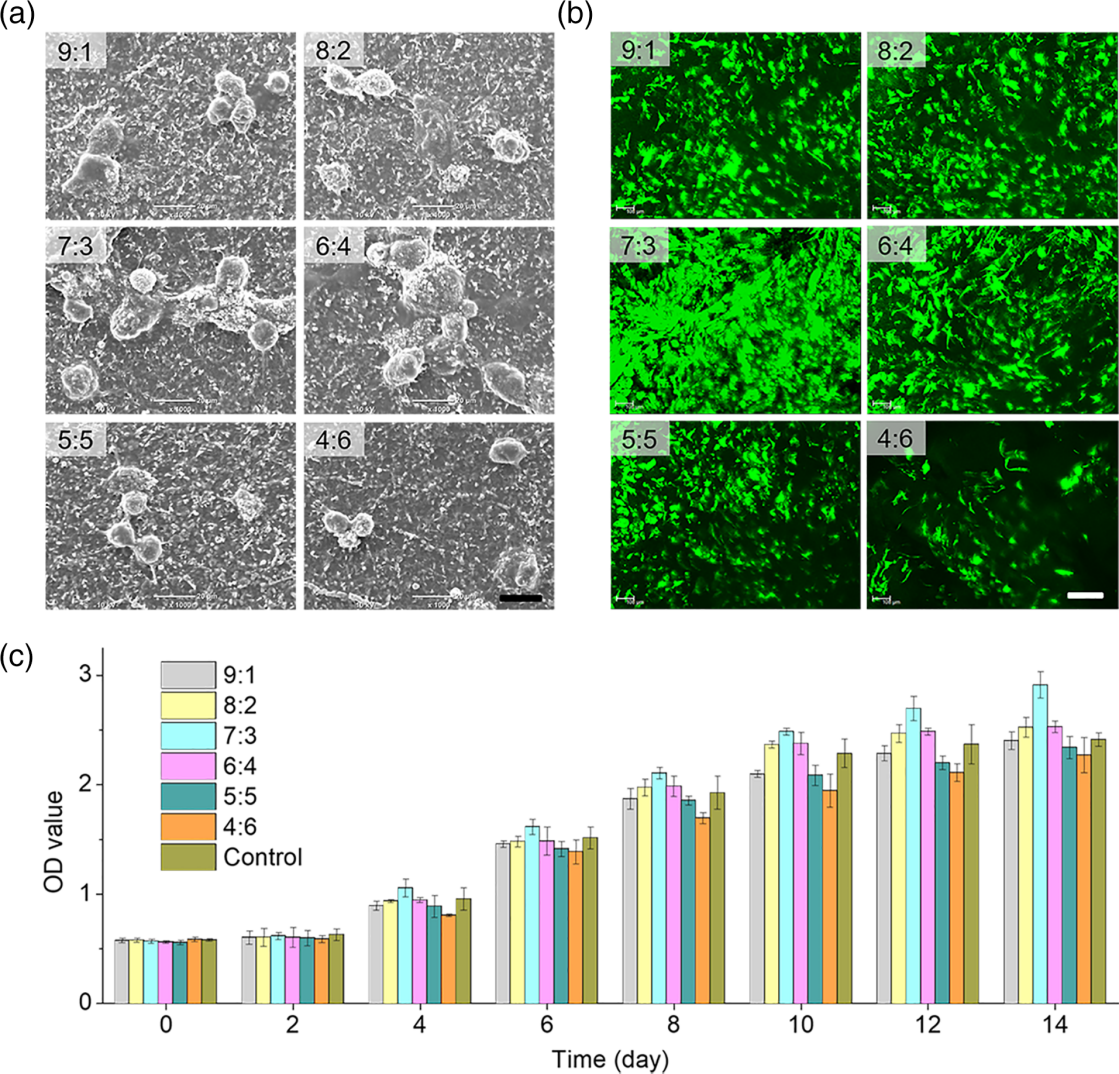
Adhesion and proliferation of adipose‐derived stem cells (ADSCs) on nanofibrous membrane (NFM) with different gelatin/chitosan proportions. (a) Spreading of ADSCs on different NFM after 3 days cultivation was observed by scanning electron microscope (SEM). Scale bars: 20 μm. (b) Morphology of ADSCs on different NFM after 14 days cultivation observed by confocal laser scanning microscope. Scale bars: 200 μm. (c) Cell proliferation on different NFM was tested by CCK‐8

Besides mechanical performances, the biocompatibility and bioactivity of NFM are also vitally important for supporting cell sheets on the surface. Therefore, the effect of gelatin/chitosan proportions on the adhesion, spreading, and proliferation behaviors of ADSCs was further investigated. At fixed time points (1, 3, and 7 days), the adhesion and spreading of ADSCs on the surface of NFMs were observed by fluorescence and SEM imaging. From Figure 2a and Figure S5a, more and more ADSCs were adhered, well‐grew, and extensively spread on the surface of all the NFMs with the increase of incubation time, and the ADSCs adhesion rate increased firstly and then decreased with the increase of chitosan content, achieving the maximum at the gelatin/chitosan proportions of 7:3 and 6:4. It can be because high surface area favors cell adhesion at the low gelatin/chitosan proportion,^13^ but gelatin has higher cell compatibility than chitosan. Therefore, the moderate gelatin/chitosan proportion will achieve highest cell adhesion. In addition, after incubation for 3 days, the shape of ADSCs on NFMs changed from round to elongated, suggesting good spreading of adhered ADSCs. After 1 week, ADSCs almost covered the whole NFMs with gelatin/chitosan proportions of 7:3 and 6:4 (Figure S5a,b).

Furthermore, effect of gelatin/chitosan proportions on the proliferation of ADSCs on the surface of NFMs was evaluated by fluorescence imaging and quantitative analysis using the CCK‐8 test. From Figure 2c, ADSCs can well proliferate on the surface of all the investigated NFMs, but the NFM with the gelatin/chitosan proportion of 7:3 caused the highest proliferation speed of ADSCs, which was further confirmed by confocal observation (Figure 2b). Moreover, the whole thickness of cell‐adhered NFM and the number of cells inside NFMs were quantified. From Figure S5c,d, it was found that cells on the gelatin/chitosan = 7:3 NFM migrated deepest inside the membrane and the corresponding thickness of cell‐adhered NFM was also highest compared to other NFMs, and the NFM with the gelatin/chitosan proportion of 4:6 showed the lowest cell migration depth. To take the overall consideration of mechanical and biological performances, the NFM with the gelatin/chitosan proportion of 7:3 was the best ideal candidate for tissue engineering in this work and was therefore selected for plasmid loading, CpMC construction, and wound‐healing experiments.

### 2.3 Sustained plasmid release behaviors of plasmid‐loaded NFM

**FIGURE 3:**
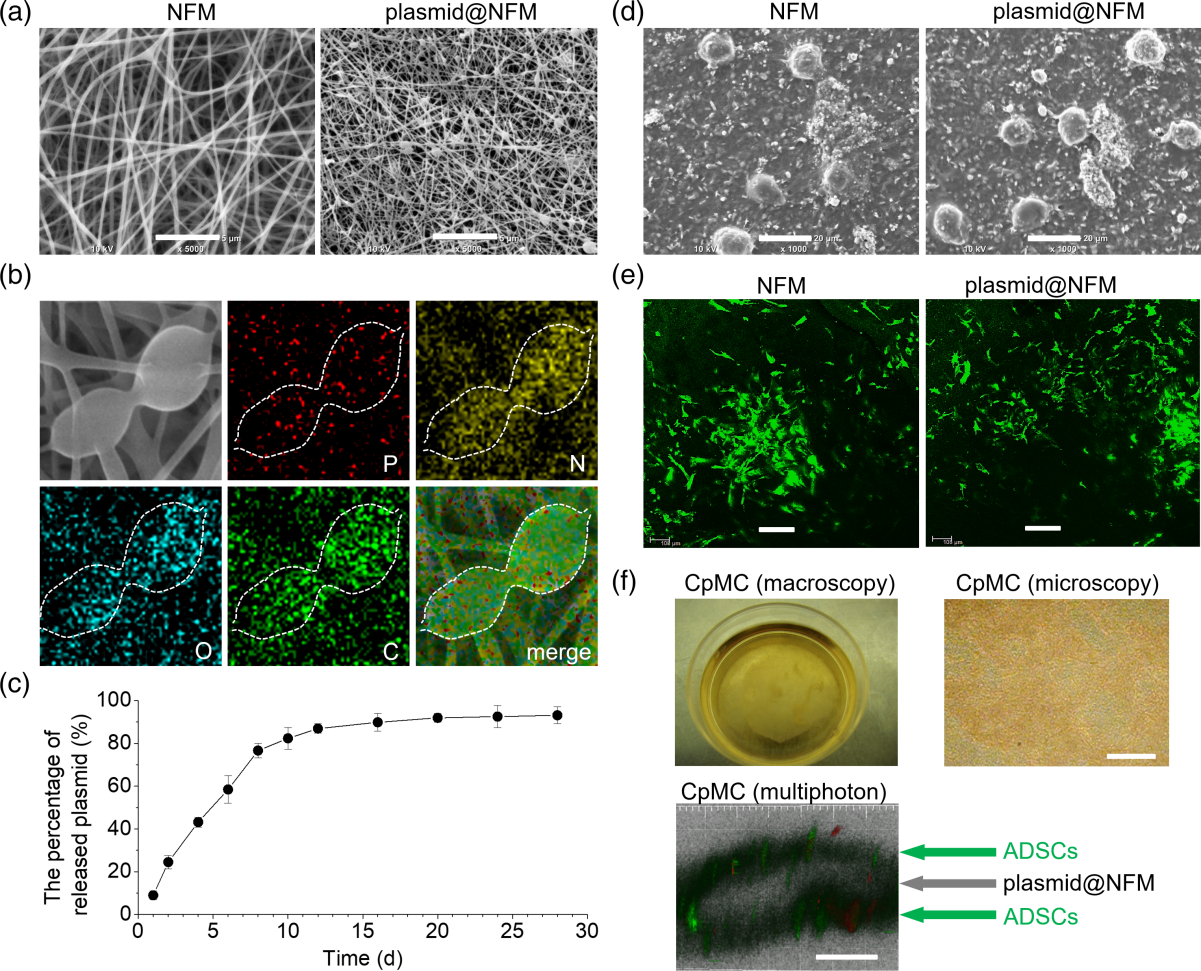
Releasing and expression properties of plasmid from plasmid@nanofibrous membrane (NFM). (a) Ultrastructure of plasmid loaded NFM observed by scanning electron microscope (SEM). Scale bars: 5 μm. (b) The compositional distribution of plasmid@NFM was further investigated by energy dispersive X‐ray spectroscopy (EDX) mapping. (c) Release profile of plasmid from plasmid@NFM. (d) Spreading of adipose‐derived stem cells (ADSCs) on plasmid@NFM after one‐week cultivation observed by SEM. Scale bars: 20 μm. (e) Proliferation of ADSCs on plasmid@NFM after 10 days cultivation observed by Confocal Laser Scanning Microscope. Scale bars: 100 μm. (f) Construction and observation of cell sheet‐plasmid@membrane‐cell sheet (CpMC) by macroscopy, microscopy, and multiphoton microscopy. Scale bars: 100 μm

The electrospinning technique enables the gelatin/chitosan NFM to be a good platform for local drug loading and drug delivery, and the water swelling and biodegradation features of NFM favor controlled drug release. VEGF plasmid was selected to be encapsulated into the nanofibers in NFM in support of angiogenesis and enhanced differentiation of ADSCs during wound healing. From SEM images in Figure 3a, different from smooth surface of NFM, there were large numbers of beads embedded within the nanofibers, which was composed of gelatin/chitosan and plasmid DNA as confirmed by elemental mapping (Figure 3b; Figure S6). Although the used VEGF plasmid had a big size (6 Kbp, about 390 nm in hydrated diameter; Figure S7), the gelatin/chitosan nanofiber with about 500 nm in diameter can still encapsulate the plasmids well, owing to electrostatic and hydrogen‐bonding attractions between gelatin/chitosan and plasmids (Figure S8), in support of successful electrospinning. Moreover, the encapsulation of VEGF plasmid did not affect the mechanical strength of NFM significantly (Figure S9). From Figure 3c, the in vitro release of plasmid in PBS showed that plasmid@NFM exhibited a sustained plasmid release behavior, in good respect to the degradation of NFM (Figure 1d). Within 2 weeks, about 90% plasmid was released from plasmid@NFM (Figure 3c). Such a release duration was expected to match with the process of wound healing. In addition, ADSCs can spread and grow very well on both NFM and plasmid@NFM (Figure 3d,e), and VEGF proteins (green fluorescence) were successfully expressed in ADSCs after ADSCs were cultured on the surface of plasmid@NFM for 2 days (Figure S10), suggesting that released VEGF plasmids were locally uptaken by ADSCs as expected (Scheme 1). Live/dead cell staining showed that released VEGF plasmids had no obvious side effects on the growth of ADSCs on the surface of plasmid@NFM (Figure S11).

Based on above encouraging results, we constructed a sandwich structure of CpMC by supporting two ADSC sheets on both sides of plasmid@NFM to investigate the effects of NFM and released VEGF plasmids on the behaviors of ADSCs for accelerating wound healing. From Figure 3f, it can be found that the sandwich structure of CpMC had been successfully constructed.

### 2.4 NFM assisted VEGF plasmid to promote the differentiation of ADSCs into endothelial, keratinocyte, and fibroblast cells

**FIGURE 4:**
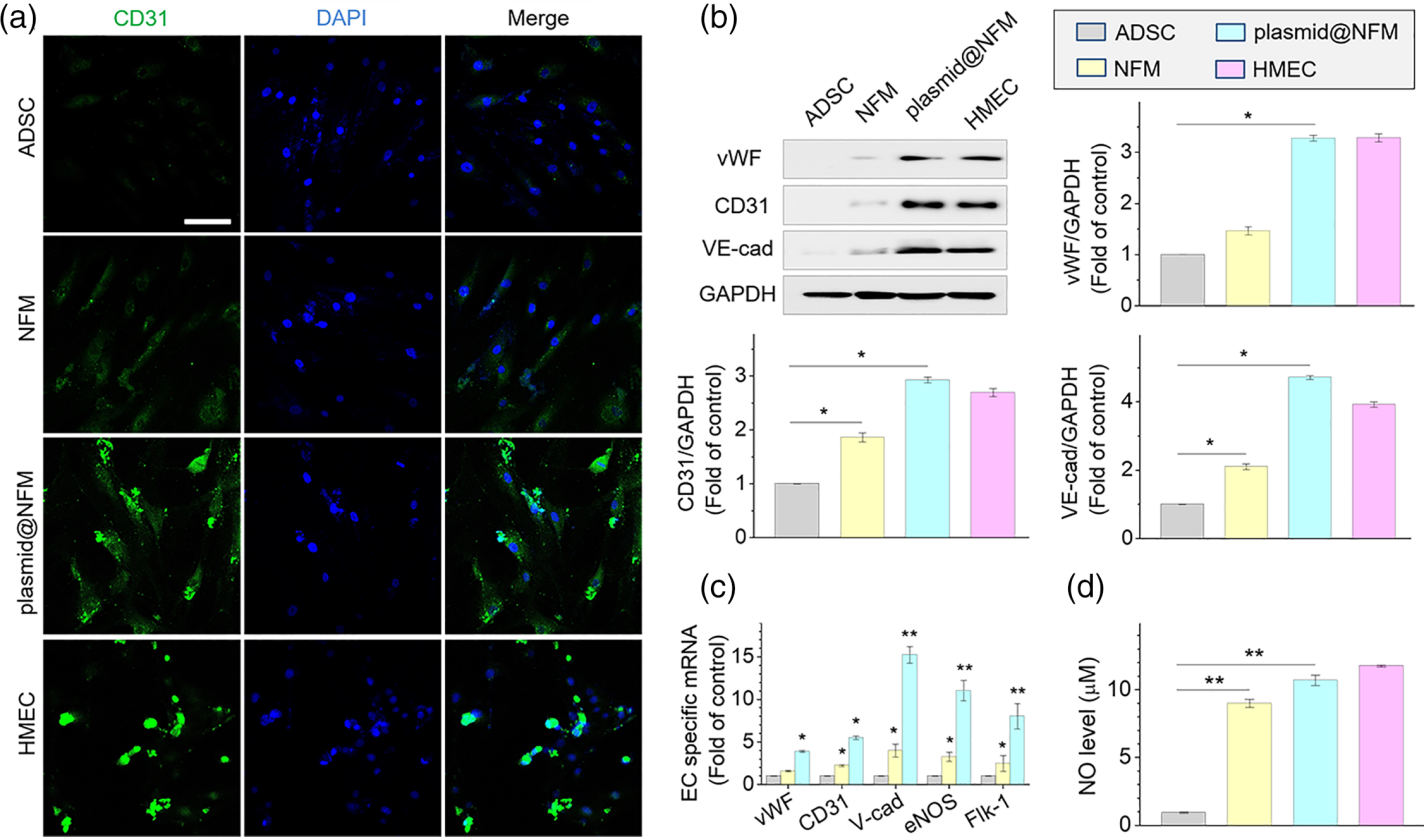
Differentiation of adipose‐derived stem cells (ADSCs) into endothelial‐like cells on plasmid@nanofibrous membrane (NFM) after 3 weeks cultivation. *p < 0.05, *p < 0.001 versus ADSC group. (a) Expression of endothelial specific marker CD31 in differentiated ADSCs tested by immunofluorescence staining; scale bar: 100 μm. (b) Expression of endothelial specific markers (vWF, CD31, and VE‐cadherin) examined by western blotting. (c) Endothelial specific genes detected by real‐time quantitative polymerase chain reaction (qRT‐PCR). (d) The concentration of NO released from differentiated ADSCs detected with DAF‐FM Diacetate. ADSC: ADSCs only (control group). NFM: ADSCs cultured on NFM. plasmid@NFM: ADSCs cultured on plasmid@NFM. HMEC, human microvascular endothelial cell as positive control

To check whether VEGF plasmid released from CpMC can promote the endothelial differentiation of ADSCs, we cultured ADSCs on the surface of plasmid@NFM for 3 weeks. From Figure S10, it can be found that differentiated ADSCs treated with NFM and plasmid@NFM showed obvious morphological changes (Figure S12), and both NFM and plasmid@NFM caused a significant increase in the transcription levels of endothelial cell‐specific markers including vWF, CD31, VE‐cadherin, eNOS, and Flk‐1 (Figure 4c). In parallel, we also observed an upregulation in the expression of specific proteins (vWF, CD31, and VE‐cadherin), whose levels in the plasmid@NFM‐treated ADSCs were almost the same with that in human microvascular endothelial cells (HMECs) (Figure 4a,b). In addition, both NFM and plasmid@NFM resulted in the remarkable increase of NO level in differentiated ADSCs (Figure 4d). Overall, released VEGF plasmids indeed promoted the differentiation of ADSCs toward endothelial cells, and the NFM matrix assisted VEGF plasmids to accelerate this process.

In order to simulate the skin microenvironment, we cocultured ADSCs separately with HaCaT cells and human skin fibroblast (HSF) cells using cell culture inserts to investigate the keratinocyte‐/fibroblast‐like differentiation and migration behaviors of plasmid@NFM‐treated ADSCs. After cocultured with HaCaT cells for 2 weeks, the morphology of plasmid@NFM‐treated ADSCs changed from fibroblast‐like to epithelioid cells as shown in Figure S13. Furthermore, NFM assisted released VEGF plasmids to promote the expressions of cytokeratin‐19, P63, and involucrin in keratinocyte‐like cells (KLCs) (Figure S14). Therein, the levels of CK19/involucrin and P63 in the plasmid@NFM‐treated ADSCs were about 6 and 70 times higher than the case of blank control (Figure S15). Differently, ADSCs still kept fibroblast‐like morphology after 2‐week coculture with HSF in spite of NFM and plasmid@NFM treatment (Figure S16). Similarly, NFM assisted released VEGF plasmids to promote the expressions of collagen I, vimentin, and S100A4 significantly (Figures S17 and S18). In a word, NFM assisted VEGF plasmids to promote the differentiation of ADSCs toward endothelial, keratinocyte, and fibroblast cells in the simulated skin microenvironment. Such a differentiation‐enhancing effect of NFM might be attributed to that gelatin as a main component of NFM is one of the ECM capable of promoting the differentiation of stem cells. In addition, we also found that VEGF can significantly promote the migration of HaCaT cells (Figure S19) and HSF cells (Figure S20), which would favor wound healing with CpMC.

### 2.5 CpMC boosted wound closure and skin regeneration

**FIGURE 5:**
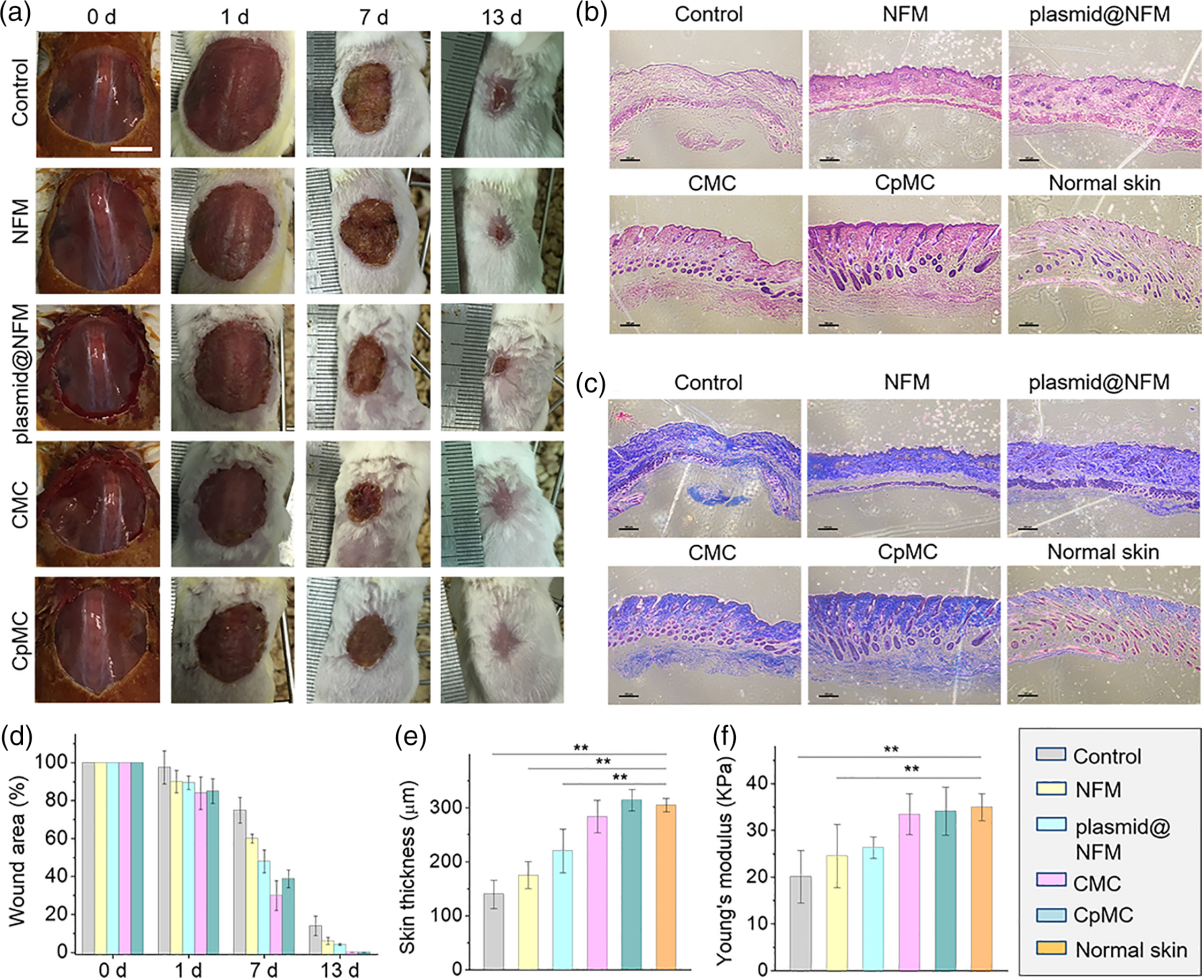
In vivo wound healing using nanofibrous membrane (NFM), plasmid@NFM, cell sheet‐membrane‐cell sheet (CMC) and cell sheet‐plasmid@membrane‐cell sheet (CpMC). (a) Images of wound beds healed using different membrane and cell sheets. (b) Histological appearance of wounds harvested on day of healing. Scale bars: 100 μm. (c) Masson's trichrome staining of wounds harvested on day of healing. Scale bars: 100 μm. (d) Changes in wound size among different group during healing. **p < 0.01. (e) Thickness of neo‐skin (epidermal and dermal layers) was measured according to histological appearance. **p < 0.01. (f) The mechanical property of neo‐skin compared with normal skin. **p < 0.01. Control: wound without transplantation; NFM: transplantation of NFM alone; plasmid@NFM: transplantation of plasmid@NFM; CMC: transplantation of CMC; CpMC: transplantation of CpMC

A large‐area wound model was built by excising a piece of 2‐cm‐diameter round full‐thickness skin on the dorsum of each mouse to investigate the wound‐healing capability of CpMC. Fresh wound was covered with CpMC and surgical dressing in turn followed by wrapping with bandage. Wound closure was assessed by macroscopic observation and calculation of the planimetric area of unclosed wound. Cell sheets with NFM have strong mechanical property and CpMC can easily be transplanted in the wound site with the preservation of membrane integrity (Figure S21), owing to high strength (Figure 1b). After transplantation for 3 days, a significant and increasing difference in wound area (*p* < 0.05) was observed in both CMC and CpMC groups compared to blank control and NFM groups (Figure 5a,d). It can be found that NFM did not affect wound closure significantly, but both plasmid@NFM and CMC had a little help and CpMC made the largest contribution to promote wound closure, owing to synergistic effect of ADSCs and VEGF plasmids. The wounds transplanted with nothing, NFM, plasmid@NFM, CMC, and CpMC were completely closed at day 19, 17, 16, and 13, respectively (Figure S22). Such a difference in the healing rate could become more distinct in the case of human wound because the skin shrinking capability of mice is much higher than that of human body.

To evaluate the wound‐healing outcomes, the primary structure and functions of new skins (neo‐skins) were examined. The thickness of neo‐skins at the day of healing including the epidermal and dermal layers was measured in H&E histological sections (Figure 5b). Masson's trichrome staining of corresponding neo‐skins was carried out at the same time to show the nascent collagen (blue staining in Figure 5c). Quantitative analysis demonstrated that the overall thickness of neo‐skin in the CpMC group was significantly thicker than that in the other groups (Figure 5e), and the matrix density increased significantly with the amount of collagen (Figure 5c). Interestingly, CpMC most remarkably increased the thickness of neo‐skin compared to the other groups, approximately achieving the level of normal skin (Figure 5e). Compared with blank control, neither NFM nor plasmid@NFM improved the fibrosis of wound and the generation of cutaneous appendages, but CMC slightly improved that and CpMC made the largest contribution especially to the generation of cutaneous appendages. Furthermore, the strengths of neo‐skins in the CMC and CpMC groups were significantly higher than that in the other groups, and were similar to that of normal skin (Figure 5f). These results indicated that the neo‐skin regenerated with CpMC exhibited excellent structure and functions similar to normal skin.

**FIGURE 6:**
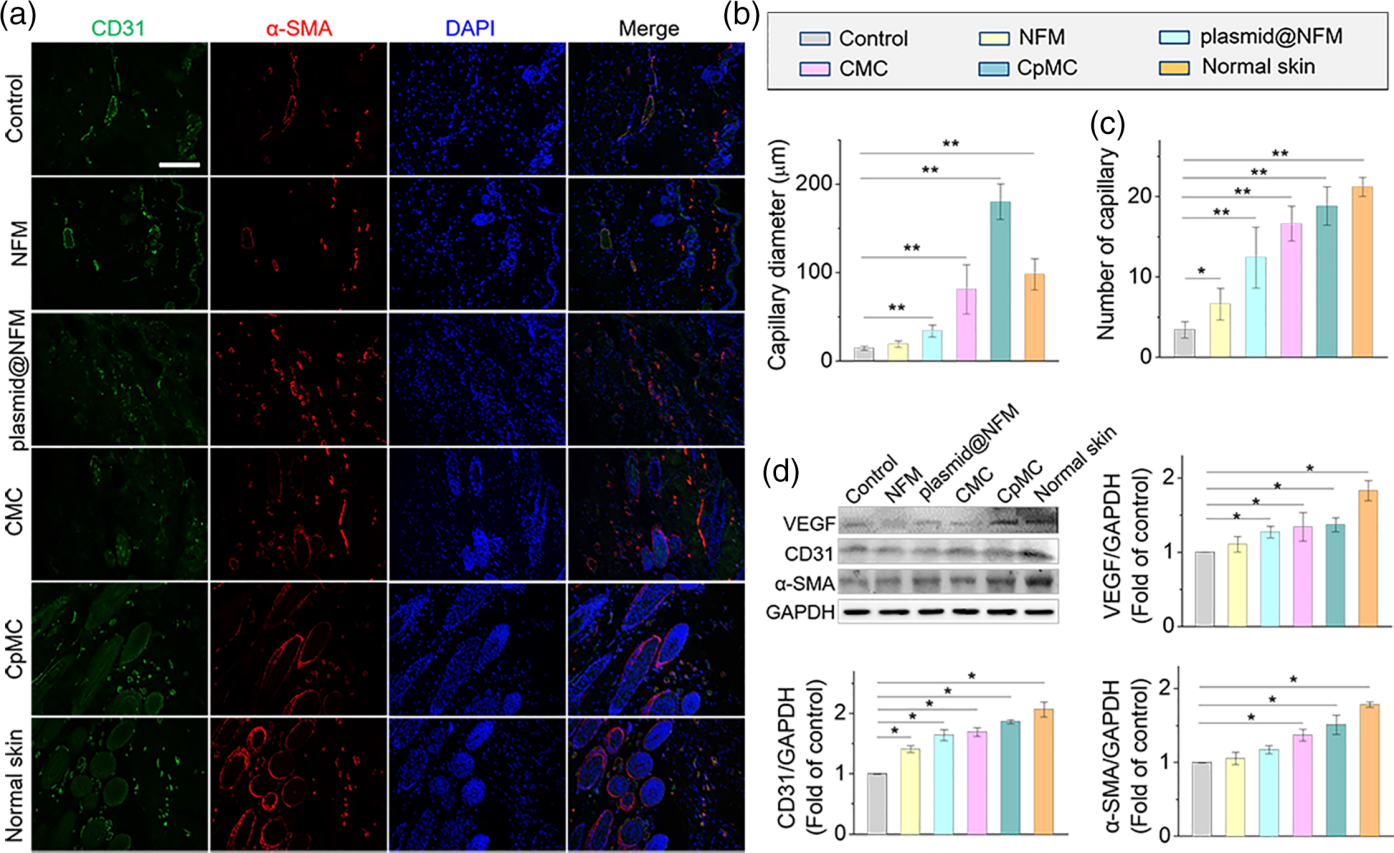
Angiogenesis examination of neo‐skins at the day of healing. (a) Immunofluorescent staining of CD‐31 (green) and α‐SMA (red) at the wound area after healing. Scale bar: 200 μm. Blue color indicates the nuclei. (b) Quantified data of average diameter of capillary at the wound area with different treatments. **p < 0.01. (c) Quantified data of capillary number at the wound area with different treatments. *p < 0.05, **p < 0.01. (d) Protein expressions (VEGF, CD31, α‐SMA) in the wound bed at the day of healing by western blot analysis. *p < 0.05, **p < 0.01

Moreover, the impact of CpMC on angiogenesis was investigated to explore the mechanism for wound healing. The blood vessels were detected by double immunofluorescence labeling of CD31 (cluster of differentiation 31) and α‐SMA (α‐smooth muscle actin). From Figure 6, compared with NFM, plasmid@NFM more significantly induced the increases in both the capillary diameter (Figure 6b) and number (Figure 6c) and the angiogenesis‐specific indicators including CD31, α‐SMA, and VEGF (Figure 6d; Figure S21), owing to the angiogenesis‐accelerating effect of VEGF plasmid in the wound site. Moreover, individual CMC also exhibited obvious acceleration of angiogenesis, due to the NFM‐induced endothelial differentiation of ADSCs (Figure 4). By comparison, CpMC displayed the strongest angiogenesis‐accelerating capability, suggesting the combined effect of VEGF plasmids and cell sheets. After wound healing with CpMC, these angiogenesis‐related parameters of neo‐skins approximated to that of normal skin, which was possibly the main reason for excellent regeneration outcomes.

## 3 DISCUSSION

In this study, we constructed a novel sandwich structure of CpMC by supporting two ADSC sheets on both sides of VEGF plasmid@NFM for efficiently repairing wound. The electrospinning technique we used can produce porous NFM with large specific growth surface, which allowed for cell proliferation and functionalization. The architecture of our electrospinning NFM made from natural biodegradable and biocompatible materials (gelatin and chitosan) was similar to the ECM of skin tissue, which can satisfy physiological requirements. However, chitosan is a hard electrospinning biomaterial, and the strength of electrospinning chitosan fiber is too weak to form nanofibers,^14^ while gelatin is too unstable and subject to biodegradation.^15^, ^16^ In this work, the blending of gelatin with chitosan can increase the spinnability of chitosan and improve the mechanical properties of chitosan fibers. Other researchers also used them to develop nanofibers,^17^, ^18^ but they did not make clear the effect of the gelatin/chitosan proportion on the physicochemical and biochemical properties of gelatin/chitosan NFM. Therefore, we fabricated the gelatin/chitosan NFMs with different proportions of gelatin and chitosan.

Our results showed that the physicochemical properties and biocompatibility of NFMs changed with the variation of gelatin/chitosan proportion. The microstructure of gelatin/chitosan NFMs clearly demonstrated that gelatin concentration was very important in obtaining fine gelatin/chitosan nanofibers, and more gelatins achieved bigger pore size, higher water absorption/swelling, and higher mechanical strength. The mechanical property of skin is important to its structure, appearance, and functionality,^19^ so the mechanical strength of NFM is also important for the skin substitute construction and wound healing. The cross‐linking treatment of gelatin/chitosan NFMs with glutaraldehyde can avoid their fast degradation.^12^ The biodegradation property of NFM was beneficial for skin regeneration as it can support and regulate skin regeneration.^15^ Gelatin absorbed an amount of water and made the NFM more hydrophilic and sensitive to degradation, while more chitosans exhibited slower degradation.^20^ Additionally, the degradation products of gelatin and chitosan are relatively nontoxic small molecules, which can easily be excreted directly or after entry and exit from various metabolic pathways.^15^, ^21^ So, all the investigated NFMs had good biocompatibility with ADSCs. ADSC is a promising adult stem cell for clinical therapy; our previous study has indicated that ADSC can be easily isolated from adipose tissue and has strong proliferation and differentiation ability.^22^ It was reported that both fiber diameter and pore size of NFM can affect cell proliferation and differentiation.^23^ The NFM with the gelatin/chitosan proportion of 7:3 exhibited appropriate fiber diameter and pore size as well as good mechanical and swelling/biodegradation properties, and thus caused most ideal ADSC adhesion and proliferation.

The gelatin/chitosan NFM was positively charged owing to amino groups, and thus negatively charged plasmids can be bound to the NFM by electrostatic attraction, which can protect plasmid from the destruction caused by enzymes and other harmful factors.^24^ So the NFM could be a promising nonviral vector for gene transfection, and enhance the formation of engineered tissue.^25^ Because of good swelling/biodegradation property of the 7:3 gelatin/chitosan NFM, we chose it for the VEGF plasmid loading. The release of VEGF plasmid DNA was unlikely to be simple diffusion but mediated by biodegradation. The sustained biodegradation/release behaviors ensured a long‐term skin repairing.

The use of biodegradable plasmid@NFM as supporting membrane was quite easy to construct and transplant CpMC for wound healing, because our previous study suggested that ADSC sheet was too thin and fragile so that it was hard to transplant intact cell sheet.^26^ A large‐area (2 cm in diameter) full‐thickness cutaneous defect in mouse was used to investigate the skin regeneration potential of CpMC. Complete wound closure and re‐epithelialization was firstly observed after implantation of CpMC for 13 days, much faster than the other transplantation. Although NFM only just had a little beneficial effect on skin regeneration, highly porous network structure and good swelling property of NFM can provide a moisture environment and enhance ADSC activity. So, both NFM and ADSCs were key elements for wound healing and skin regeneration in this work. The wound skin transplantation of CpMC had thicker and complete epithelial layer than that of CMC, which indicated that VEGF also played an important role in wound healing and skin regeneration.

VEGF is a multitasking cytokine which stimulates cell survival, proliferation, migration, and differentiation,^27^ which is also confirmed in this work. VEGF released from CpMC locally promoted the differentiation of ADSCs into endothelial, keratinocyte, and fibroblast cells. Interestingly, NFM also had a little effect on the differentiation of ADSCs. One of possible reasons may be that gelatin, as a denatured form of collagen and one component of the ECM, can promote the differentiation of stem cells in specific microenvironment.^28^, ^29^ Besides, VEGF also can enhance the migration of keratinocyte and fibroblast cells, which may be beneficial for the wound repairing, because the whole wound‐healing process includes cell adhesion, proliferation and migration, and eventually leads to skin regeneration.^30^

Endogenous angiogenic factor, VEGF, is naturally produced in response to tissue hypoxia during wound healing.^31^ Because of inadequate secretion of VEGF, this restorative process of wound is insufficient to prevent tissue ischemia and necrosis. During wound healing, rebuilding damaged blood vessels (angiogenesis) is extremely important since repairing activities are highly dependent on newly formed blood vessels to supply oxygen and nutrients.^32^ Wound treated with CpMC showed larger diameter of capillary, indicating more oxygen and nutrients supply for skin tissue regeneration. VEGF plays an important role in endothelial cell differentiation and vascular repair.^33^ The enhanced expressions of CD31 and α‐SMA, two widely used markers for blood vessels,^34^ implied that the CpMC‐mediated VEGF expression promoted endothelial cell differentiation and accelerated the maturation of blood vessels to accommodate the urgent demand of oxygen and nutrients for wound regeneration. It is well known that angiogenesis is modulated by VEGF.^35^ Our results suggested that CpMC facilitated angiogenesis and skin cell differentiation as well as migration.

## 4 EXPERIMENTAL SECTION

### 4.1 Preparation of electrospun gelatin/chitosan NFM

Fifteen percent gelatin (from bovine skin, type B, Sigma) and 1% chitosan (low molecular weight, viscosity: 20–300 cp, Sigma) were suspended in hexafluoroisopropanol (Sigma) separately to form solutions. To this, stirred suspension chitosan (1% w/v) and 15% (w/v) of gelatin solution were added with different proportion (15% gelatin: 1% chitosan, 1:9, 2:8, 3:7, 4:6, 5:5, 6:4, 7:3, 8:2, 9:1). The mixture was stirred at room temperature overnight. Electrospinning was done at a voltage of 8 kV to create micro/nanofibers with a needle having an inner diameter of 20 gauge and a 0.6 ml/h feeding rate of solution using a syringe pump. The cellophane collector plate was placed 12 cm away from the tip of the needle. Upon the completion of the electrospinning, the electrospun NFM were removed from the collector and cross‐linked with glutaraldehyde for 1 h.

For the impregnation of plasmid DNA into NFM, 2 mg of plasmid DNA pCMV6‐AC‐VEGF (500 μg/μl, vector pCMV6‐AC‐GFP was bought from ORIGENE, Beijing) were gently mixed with gelatin/chitosan electrospun solution, and electrospinning was done as described. The size of pIRES2‐AcGFP1 vector is 5.3 kbp. Human VEGF DNA fragment (699 bp) was inserted into Sgf1 and Mlu1. The charge ratio (N:P) that is indicated as the molar ratio of the free amino groups of gelatin/chitosan NFM to the phosphate molecules of plasmid DNA is 5.0, according to our previous results, to obtain the highest transfection efficiency.^24^ The NFM with plasmid (plasmid@NFM) also need to be cross‐linked with glutaraldehyde to avoid fast‐degrading.

### 4.2 Characterization of electrospun NFM

#### 4.2.1 Microstructure of NFM by SEM

The morphology of the NFM was studied by SEM (JCM 6000 JEOL, Japan). The samples were sputter coated, placed on SEM holders, and an accelerating voltage of 10 kV was utilized for imaging. Using image processing software (ImageJ), the average parameter of 100 pores and the diameters of 100 electrospun fibers were measured from the obtained SEM images.

#### 4.2.2 Degradation property

NFM were placed in a 24‐well plate with 2 ml PBS (pH 7.4) and kept at 37°C for degradation time periods of *T* = 5, 10, 15, 20, 25, 30, and 35 days (*n* = 5 samples at each time point). An additional time point of *T* = 0 days refers to nondegraded reference samples. Since the non‐cross‐linked NFM is fast‐degrading (degraded in PBS within half an hour, data did not show), it is not suitable for the construction of multilayer cell sheets, and only cross‐linked NFM was examined. After degradation, the samples were removed from PBS and dried in a vacuum (YIHENG17, Shanghai, China) at room temperature for 90 min. The remaining weight percentage of NFM was calculated according to the following equation: Residual mass (%) = *W*_t_/*W*_0_ × 100% = *W*_t_ *W*_0_ × 100%. Where *W*_0_ is the initial weight of the NFM and *W*_t_ is the remaining weight of the NFM at each time point. The pH of the degradation fluid of each sample was measured at each time point (METTLER TOLEDO SevenGo pH meter SG2, Switzerland).

#### 4.2.3 Swelling property

Cross‐linked NFM were submerged in PBS at 37°C for 24 h. Swelling degree was then measured by the formula: Degree of swelling (%) = [(*M* − *M*_d_) /*M*_d_] × 100. Where *M* is the weight of each sample after immersion in the PBS for 24 h and *M*_d_ is the weight of the sample in its dry state. Only cross‐linked NFM was examined.

#### 4.2.4 Mechanical property

The NFM were collected using cellophane, and then the thickness of each sample was measured by SEM. These samples were measured, cut carefully and glued on the frame, and then the cellophane was removed. The paper frame was cut before initiating the tensile strength measurements. The tensile properties of these samples were characterized by a tensile testing machine (UTM150, Advanced Nanomeasurement Solutions, Agilent Technologies). The velocity was 100 μm/s. The data were analyzed by using the Linksys32 software. The reported Young's modulus represented average results of the five samples.

#### 4.2.5 Furrier transform‐infrared spectroscopy

The functional changes in different NFM were evaluated using Thermo Scientific TM Nicolet‐6700 FTIR (Shanghai, China). For this, each non‐cross‐linked and cross‐linked nanofiber were scanned from 4000 to 400/cm with step resolution of 0.4 and 32 number of scans.

#### 4.2.6 Differential scanning colorimetry (DSC)

DSC was carries out on the DSC Q200 differential scanning colorimeter (TA Instrument, Delaware, USA). Each sample was analyzed between 25°C and 300°C with 5°C/min of heating rate. Nitrogen gas was used as inert gas and slandered aluminum crucibles were used for experiment.

#### 4.2.7 Release profile of plasmid DNA from plasmid@NFM

Sustained release of plasmid DNA from plasmid@NFM was measured by Microplate Reader (Epoch2, Biotech, USA). Phosphate buffer saline (pH 7.4) was used for in vitro plasmid release studies. Three plasmid@NFM were cut into small equally sized pieces separately. A 1 mg of plasmid@NFM was analyzed for the release studies at 37°C and stirring at 100 rpm. A 1 μl of aliquots of each plasmid loaded samples was assayed at predetermined regular time intervals: 0, 1, 2, 4, 6, 8, 10, 12, 14, and 16 days, five aliquots of each sample were done at each time point. The release profiles were plotted as a function of time in terms of cumulative percentage plasmid released.

### 4.3 Biocompatibility of different NFM and plasmid@NFM with stem cell

NFM 10 mm in diameter were sterilized with 70% ethanol (30 min), and then washed with PBS and suspended in culture medium (15 min). Human ADSCs were isolated according to our previous method^22^ (human subcutaneous adipose tissue was obtained from subjects undergoing surgery with informed patient consent, and the study was approved by the Medical Ethical Committee and the Animal Ethical and Welfare Committee of Shenzhen University), and cultured in DMEM with 10% FBS, 5 × 10^4^ cells/ml ADSCs at passage 4 or 5 were used in the following experiment. ADSCs were transfected with green fluorescent protein (GFP) by adenovirus vector to obtain ADSCs‐GFP. ADSCs‐GFP were cultured on NFM. The same cell number was seeded on different NFM and cultured at 37°C in a humidified air atmosphere with 5% CO_2_. For the VEGF protein expression, ADSCs without GFP were seeded on the NFM, because the plasmid vector pCMV6‐AC‐GFP can expression GFP, and VEGF proteins will show green under confocal laser scanning microscope after expression in ADSCs. After 3 days of culture, cellular constructs were harvested and fixed with 2% glutaraldehyde for 15 min and washed with PBS two times (15 min/time). The samples were then dehydrated through a series of graded ethanol solutions (50%, 75%, 90%, 95%, and 100%) and 5 min was used for every washing time. The cells were then washed three times in 100% ethanol. All samples were air‐dried overnight. Dry cellular constructs were sputtered with gold and imaged by SEM.

After 2 weeks of culturing, ADSCs on NFM were washed once with PBS, and confocal laser scanning microscope (Leica Microsystems) was used to observe cells on NFM. Cell migration was quantified using the whole thickness of cells, defined as the average depth of cell layers detected by confocal laser scanning microscope.

To test cell proliferation on the gelatin/chitosan NFM, we seeded the cells at the same density and incubated the samples for different time periods. The prepared NFM were cut according to the diameter of the well in a 96‐well flat bottom plate. Each of the 96 wells contained NFM adjusted separately at the bottom of each well under aseptic conditions. The NFM were sterilized with 70% ethanol followed by washing with PBS and culture medium. Culture medium containing cells suspension at a density of 5 × 10^4^ cells/ml was added to each well. Wells devoid of NFM were filled with same amount of cell suspension used as control. After the incubation period, 10 μl of sterile cell counting kit‐8 were added to each well and incubated for 3 h at 37°C. The percentage of viable cells was determined every day for 15 days. The optical density values were examined at a test wavelength of 450 nm and reference wavelength of 630 nm, and at least in triplicate against a reagent blank.

To test the expression of VEGF in ADSCs, ADSCs without GFP were seeded on plasmid@NFM, VEGF protein expression in the ADSCs was observed by confocal laser scanning microscope (Leica Microsystems). For the biocompatibility of plasmid@NFM with ADSCs, ADSCs‐GFP were cultured on plasmid@NFM for 1 to 2 weeks and observed with SEM (JCM 6000 JEOL, Japan) and confocal laser scanning microscope (Leica Microsystems).

### 4.4 Differentiation of ADSC into endothelial‐like cell, KLCm and fibroblast‐like cell on plasmid@NFM

To differentiate ADSC into endothelial‐like cells, ADSCs were seeded on NFM or plasmid@NFM and cultured with culture media (DMEM contain 10% FBS) for 3 weeks. The specific proteins of endothelial cell CD31, VE‐cadherin, and vWF were detected by immunofluorescent staining and western blot; NO released from endothelial cells were detected by ultraviolet spectrophotometer. The expression of endothelial cell specific genes (vWF, CD31, VE‐cadherin, eNOS, and Flk‐1) was detected by real‐time quantitative polymerase chain reaction according to our previous method,^20^ the primers were showed in Table S1. HMEC was used as positive control. To differentiate ADSC into KLC, we used the cocultured system as previously described.^36^ ADSCs and HaCaT cells were inoculated in the two chambers separated by cell culture inserts (CORNING, USA). HaCaT cells were in the upper chamber and ADSCs seeded on NFM or plasmid@NFM in the lower. Complete media (DMEM contain 10% FBS) were added for 2 weeks. ADSCs only served as the control group. The specific proteins of keratinocyte were examined by immunofluorescent staining and western blot. The same coculture method was used to differentiate ADSCs into fibroblast‐like cells; HSF was seeded in the upper chamber to provide the differentiation media.

To investigate the effects of VEGF on the migration of HaCaT and HSF, transwell cell culture insert was applied and cultured for 24 h. Wound‐healing assay was also applied to test the migration ratio of HaCaT cells.

### 4.5 Preparation and transplantation of CpMC

According to the physical characteristics and biocompatibility with ADSC, we chose gelatin/chitosan (7:3) NFM or plasmid@NFM to support ADSC sheets. After culturing ADSCs on the surface of temperature‐responsive culture dish to a confluent layer, all media were aspirated and fresh media were added to prevent the cells from drying out. The NFM or plasmid@NFM was gently placed on top of the cell layer and incubated at 20°C for 20 min. Forceps were used to grasp under the membrane and cell layer, and carefully withdraw them from the surface. The membrane, with the attached cell layer facing downward, was transferred to another cell layer on the thermoresponsive cell culture surface and incubated at 20°C for 20 min, and then added media and cultured for 24 h; the detached CMC or CpMC sandwich‐like cell sheets were used for transplantation.

All animals were treated in compliance with the Guide for the Care and Use of Laboratory Animals (NIH Publications No. 8523, revised 1996). The protocol was approved by Animal Ethical and Welfare Committee of Shenzhen University (SYXK: 2018‐0140). All efforts were made to minimize suffering and numbers of mice used. Specific pathogen‐free kunming mice were purchased from Guangdong Medical Laboratory Animal Center, weighting 32 ± 2 g, and were anesthetized in a chamber of 2% isoflurane (Jinan Shengqi Pharmaceutical Co, China) with 40% oxygen and 60% nitrous oxide. After shaving, one excision of 2‐cm diameter round shape was performed in the dorsum of each animal. Wounds were assigned randomly to the following groups (*n* = 6 wounds for each group): (1) empty wound (control), (2) 7:3 gelatin/chitosan NFM alone (NFM), (3) 7:3 gelatin/chitosan NFM with VEGF plasmid (plasmid@NFM), (4) CMC, and (5) CpMC. The membrane and cell sheets were transplanted and covered on the wound; the implanted membrane and cell sheets adsorbed on wounds and would not be dislocated. All wounds were covered and bandaged with surgical dressing. Animals were kept in individuals' cages with food and water ad libitum, and observed daily during the total period of the experiment. At the day of wound healing, animals were euthanized by isoflurane inhalation and the regenerated skins were excised out and collected at the established end points, froze in liquid nitrogen, or fixed in 4% paraformaldehyde and paraffin‐embedded. Representative sections were stained for hematoxylin & eosin (H&E) and Masson's trichrome following routine protocols. Sections were analyzed and images were acquired with an optical microscope, Olympus BX‐41/Q‐Color3 digital camera (Olympus, Japan).

### 4.6 Immunofluorescence staining and western blotting

For the tissue section, after deparaffinization, hydration, and blocking of the nonspecific binding sites with 5% bovine serum albumin (BSA) at 37°C for 30 min, tissue sections were incubated with a rabbit polyclonal antibody to CD31 (1:200, ab28364, Abcam), a mouse polyclonal antibody to α‐SMA (1:100, ab7817, Abcam), and a rabbit polyclonal antibody to VEGF (1:100, ab28364, Abcam) at 4°C overnight. Then, sections were washed with PBS, followed by incubation with a donkey anti‐rabbit IgG Alexa Fluor® 488‐conjugated secondary antibody (1:1000, ab150073, Abcam) and a donkey anti‐mouse IgG Alexa Fluor® 647‐conjugated secondary antibody (1:1000, ab150111, Abcam) at 37°C for 60 min in the dark. After being stained with DAPI and mounted with anti‐fluorescence quenching reagent, the tissue sections were observed and imaged with a Nikon confocal laser microscope (Nikon, A1 PLUS, Tokyo, Japan). For the immunocytochemistry, the cells were fixed with 4% formaldehyde, and incubated the primary and secondary antibody according to the above procedure.

Total protein was obtained by lysing tissues or cells in radioimmunoprecipitation assay buffer containing protease and phosphatase inhibitors. Following mixing with loading buffer and boiling, the prepared samples were separated on 10% or 8% SDS‐PAGE gels and transferred onto polyvinylidene fluoride membranes (Millipore, CA, USA). After blocking with 3% BSA, the membranes were incubated overnight at 4°C with the following primary antibodies: a rabbit polyclonal antibody to CD31 (1:200, ab28364, Abcam), a mouse polyclonal antibody to α‐SMA (1:100, ab7817, Abcam), and a rabbit polyclonal antibody to VEGF (1:100, ab28364, Abcam), or mouse‐anti GAPDH (Abcam, 1:2000, ab8226). The membranes were then washed in TBS‐Tween 20 and incubated with horseradish peroxidase‐coupled anti‐rabbit or anti‐mouse antibodies (KPL, MD, USA), and were detected by chemical luminescence and visualized on a luminescent image analyzer (ImageQuant LAS4000mini, Sweden). Densitometric analyses were performed using Gel‐Pro Analyzer 4.0 software (Media Cybernetics, Rockville, USA).

### 4.7 Quantitative real‐time polymerase chain reaction

Total RNA from the differentiated cells was obtained using Trizol (Invitrogen). The RNA was reverse transcribed to complementary DNA (cDNA) using the First Strand cDNA kit (Takara) following the manufacturer's protocol. Quantitative polymerase chain reaction analysis was then performed using the Quantitect SYBR Green PCR Master Mix (Takara). For amplification process, the sense and anti‐sense primers (Table S1) were used. Standard curves were generated, and quantities of each transcript were normalized to GAPDH as an internal control. Each assay was performed in triplicate with an *n* of 3 independent experiments.

### 4.8 Statistical analysis

All data were shown as the mean ± SD. All experiments were independently repeated for at least three times unless otherwise stated. Differences between groups were assessed by independent‐samples *t* test or by one‐way analysis of variance followed by Tukey's multiple comparison test. All analyses were performed in SPSS 22.0 software and *p* < 0.05 was considered to be significant unless otherwise specified.

## 5 CONCLUSIONS

In this study, we fabricated a biodegradable NFM to locally encapsulate VEGF plasmid and support multilayer stem cell sheets transplantation for wound healing. The gelatin/chitosan (7:3) NFM had appropriate biodegradation and mechanical strength, as well as good biocompatibility with ADSCs, and could release VEGF plasmid in a sustained way to promote the migration of skin cells, proliferation and differentiation of stem cells. The gelatin/chitosan NFM supported the multilayer stem cell sheets and made it easy to transplant into the wound site. This NFM can be a good plasmid delivery platform, and the proposed membrane‐supporting cell sheet strategy provides a new route to tissue engineering, and the developed CpMC demonstrates a high potential for clinical translation.

## CONFLICT OF INTEREST

All authors declared no potential conflicts of interest.

## AUTHOR CONTRIBUTIONS

**Yanxia Zhu:** Conceptualization; funding acquisition; investigation; project administration; resources; writing ‐ original draft. **Yuqi Liao:** Investigation. **Yuanyuan Zhang:** Investigation. **Mehdihasan I Shekh:** Investigation; methodology. **Jianhao Zhang:** Investigation. **Ziyang You:** Investigation. **Bing Du:** Investigation; methodology. **Cuihong Lian:** Project administration; resources; supervision. **Qianjun He:** Conceptualization; funding acquisition; project administration; supervision; writing‐review & editing.

### PEER REVIEW

The peer review history for this article is available at https://publons.com/publon/10.1002/btm2.10244.

## Supporting information


**Appendix** **S1**: Supporting information
**Figure S1**. Morphological characterization of different gelatin/chitosan NFM. Fiber diameter distribution (A) and average pore sizes (B) of NFM with different gelatin/chitosan proportions
**Figure S2**. pH value of degradation fluid in different gelatin/chitosan NFM
**Figure S3**. FTIR spectra of gelatin/chitosan NFM. FTIR spectra of non‐crosslinked (A) and crosslinked (B) gelatin/chitosan NFM
**Figure S4**. DSC thermograms of gelatin/chitosan NFM. DSC curves of non‐crosslinked (A) and crosslinked (B) gelatin/chitosan NFM
**Figure S5**. Adhesion and proliferation of ADSCs on NFM with different gelatin/chitosan proportions. (A) ADSC‐GFP adhered on the NFM after one day cultivation. (B) The number of spreading cells on different gelatin/chitosan NFM. (C) After one week culture, ADSC‐GFP almost coved the surface of NFM. Scale bars 200 μm. (D) The thickness of cell‐NFM was measured by confocal laser microscope
**Figure S6**. The composition of plasmid@NFM confirmed by energy dispersive X‐ray spectroscopy
**Figure S7**. The size of VEGF plasmid measured by DLS
**Figure S8**. The interaction of gelatin/chitosan and plasmid
**Figure S9**. The mechanical property of plasmid@NFM
**Figure S10**. The expression of VEGF protein in ADSCs cultured on gelatin/chitosan (7:3) NFM or plasmid@NFM. Scale bar 200 μm
**Figure S11**. Live/dead cell staining after one‐week culture on gelatin/chitosan (7:3) NFM or plasmid@NFM. Scale bar 200 μm
**Figure S12**. Morphological changes of ADSCs after culturing on NFM or plasmid@NFM for 3 weeks. Scale bar 100 μm. ADSC: ADSCs only (negative control). HMEC: human microvascular endothelial cell as positive control
**Figure S13**. Morphological changes of ADSCs cultured on NFM or plasmid@NFM after coculturing with HaCat cells for 2 weeks. Scale bar 100 μm. ADSC: ADSC only as negative control; coculture: ADSC coculturing with HaCat by cell culture insert; NFM: ADSC cultured on NFM coculturing with HaCat by cell culture insert; plasmid‐NFM: ADSC cultured on plasmid@NFM coculturing with HaCat by cell culture insert
**Figure S14**. Protein expression of CK19 in differentiated ADSCs cultured on NFM or plasmid@NFM after coculturing with HaCat cells for 2 weeks. Scale bar 200 μm
**Figure S15**. Keratinocyte‐specific protein expression of differentiated ADSCs cultured on NFM or plasmid@NFM after coculturing with HaCat cells for 2 weeks. *p < 0.05 versus control group
**Figure S16**. Morphological changes of ADSCs cultured on NFM or plasmid@NFM after coculturing with HSF cells for 2 weeks. Scale bar 100 μm
**Figure S17**. Protein expression of collagen I in differentiated ADSCs cultured on NFM or plasmid@NFM after coculturing with HSF cells for 2 weeks. Scale bar 200 μm
**Figure S18**. Fibroblast‐specific protein expression of differentiated ADSCs cultured on NFM or plasmid@NFM after coculturing with HSF cells for 2 weeks. *p < 0.05
**Figure S19**. Migration of HaCat cells induced by VEGF. (A) The migration of HaCat cells induced by VEGF examined by transwell inserts, migrated HaCat cells showed by DAPI staining. Scale bar 200 μm. (B) Quantitative analysis of migrated cells. *p<0.05. (C) The migration of HaCat cells tested by wound healing assay. Scale bar 200 μm. (D) Migration ratios of HaCat cells. *p<0.05
**Figure S20**. Migration of HSF cells induced by VEGF tested by transwell insert. (A) Migrated HSF cells showed by DAPI staining. Scale bar 200 μm. (B) Quantitative analysis of migrated cells. *p<0.05.
**Figure S21**. Transplantation of plasmid@NFM or CpMC onto the wound site
**Figure S22**. Wound with different treatment closed at different day
**Figure S23**. Immunofluorescent staining of VEGF (green) at the wound area after healing. Blue colour indicates the nuclei. Scale bar in all images were 100 μm
**Table S1**. Sequences of primers for RT‐PCR
**Table S2**. Characteristics of different Non crosslinked & crosslinked gelatin/chitosan NFM
**Table S3**. DSC parameters of different Non crosslinked & crosslinked gelatin/chitosan NFM.

## Data Availability

The main data supporting the findings of this study are available within the paper and its Supplementary information. The associated raw data are available from the corresponding author on reasonable request.
