# Supplementary material for: Novel nanofibrous membrane‐supporting stem cell sheets for plasmid delivery and cell activation to accelerate wound healing
Source: Bioeng Transl Med. 2021 Aug 12;7(1):e10244. doi: 10.1002/btm2.10244 (PMC8780893; doi:10.1002/btm2.10244)
Supplement: Supplementary file 1 — Appendix S1: Supporting information Figure S1. Morphological characterization of different gelatin/chitosan NFM. Fiber diameter distribution (A) and average pore sizes (B) of NFM with different gelatin/chitosan proportions Figure S2. pH value of degradation fluid in different gelatin/chitosan NFM Figure S3. FTIR spectra of gelatin/chitosan NFM. FTIR spectra of non‐crosslinked (A) and crosslinked (B) gelatin/chitosan NFM Figure S4. DSC thermograms of gelatin/chitosan NFM. DSC curves of non‐crosslinked (A) and crosslinked (B) gelatin/chitosan NFM Figure S5. Adhesion and proliferation of ADSCs on NFM with different gelatin/chitosan proportions. (A) ADSC‐GFP adhered on the NFM after one day cultivation. (B) The number of spreading cells on different gelatin/chitosan NFM. (C) After one week culture, ADSC‐GFP almost coved the surface of NFM. Scale bars 200 μm. (D) The thickness of cell‐NFM was measured by confocal laser microscope Figure S6. The composition of plasmid@NFM confirmed by energy dispersive X‐ray spectroscopy Figure S7. The size of VEGF plasmid measured by DLS Figure S8. The interaction of gelatin/chitosan and plasmid Figure S9. The mechanical property of plasmid@NFM Figure S10. The expression of VEGF protein in ADSCs cultured on gelatin/chitosan (7:3) NFM or plasmid@NFM. Scale bar 200 μm Figure S11. Live/dead cell staining after one‐week culture on gelatin/chitosan (7:3) NFM or plasmid@NFM. Scale bar 200 μm Figure S12. Morphological changes of ADSCs after culturing on NFM or plasmid@NFM for 3 weeks. Scale bar 100 μm. ADSC: ADSCs only (negative control). HMEC: human microvascular endothelial cell as positive control Figure S13. Morphological changes of ADSCs cultured on NFM or plasmid@NFM after coculturing with HaCat cells for 2 weeks. Scale bar 100 μm. ADSC: ADSC only as negative control; coculture: ADSC coculturing with HaCat by cell culture insert; NFM: ADSC cultured on NFM coculturing with HaCat by cell culture insert; plasmid‐NFM: ADSC cultured on plasmid [file BTM2-7-e10244-s001.doc]

Supporting Information

Novel nanofibrous membrane-supporting stem cell sheets for plasmid delivery and cell activation to accelerate wound healing

Yanxia Zhu^1,2^, Yuqi Liao^1^, Yuanyuan Zhang^1,3^, Mehdihasan I. Shekh^4^, Jianhao Zhang^1^, Ziyang You^1^, Bing Du^4^, Cuihong Lian^1,3*^, Qianjun He^2*^


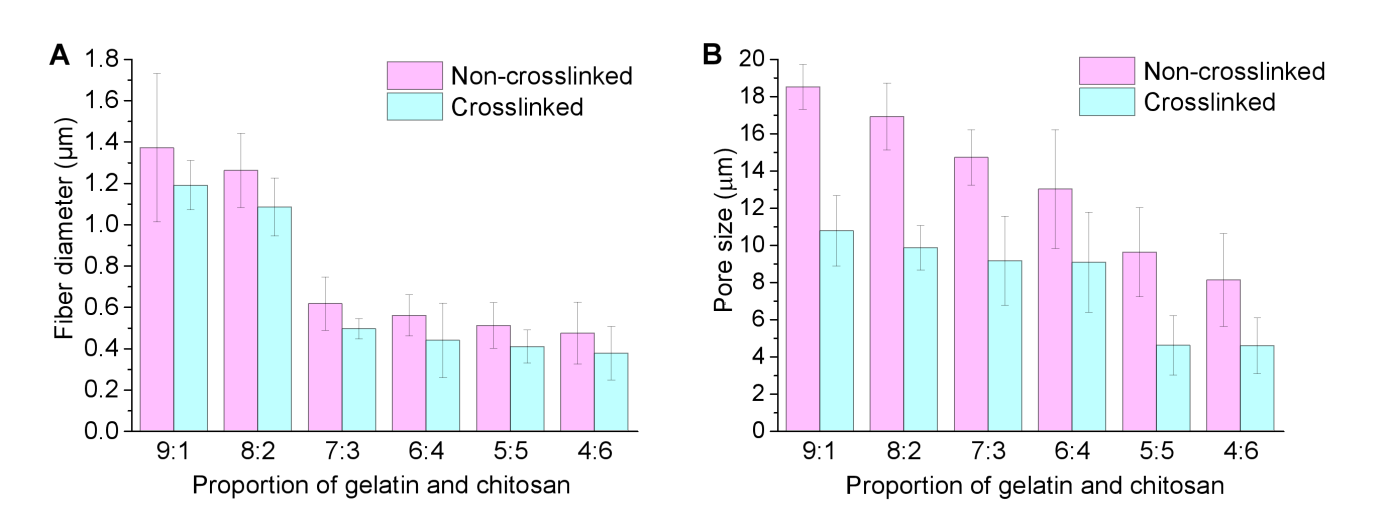


**Figure S1.** Morphological characterization of different gelatin/chitosan NFM. Fiber diameter distribution **(**A) and average pore sizes (B) of NFM with different gelatin/chitosan proportions.


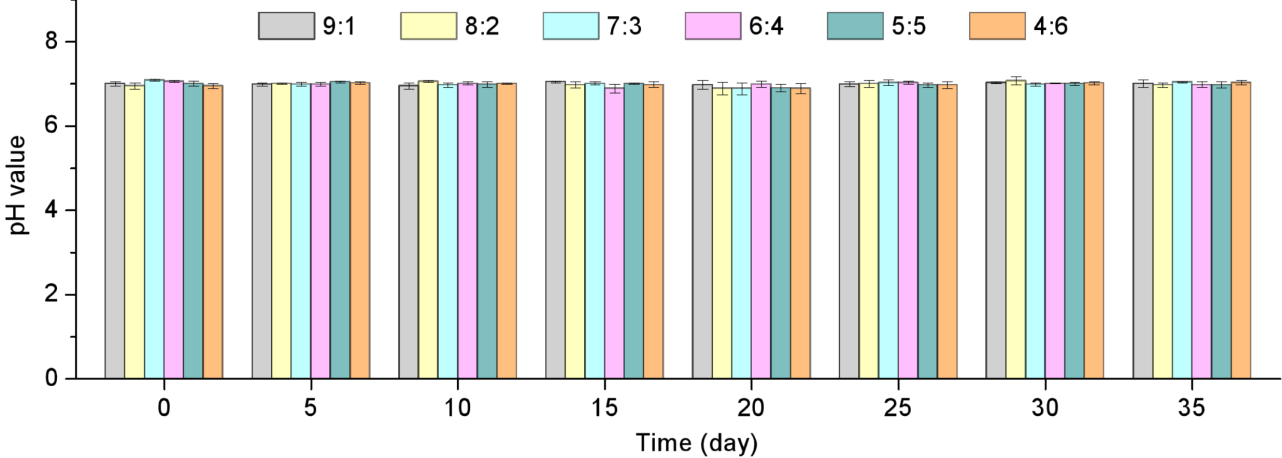


**Figure S2.** pH value of degradation fluid in different gelatin/chitosan NFM.


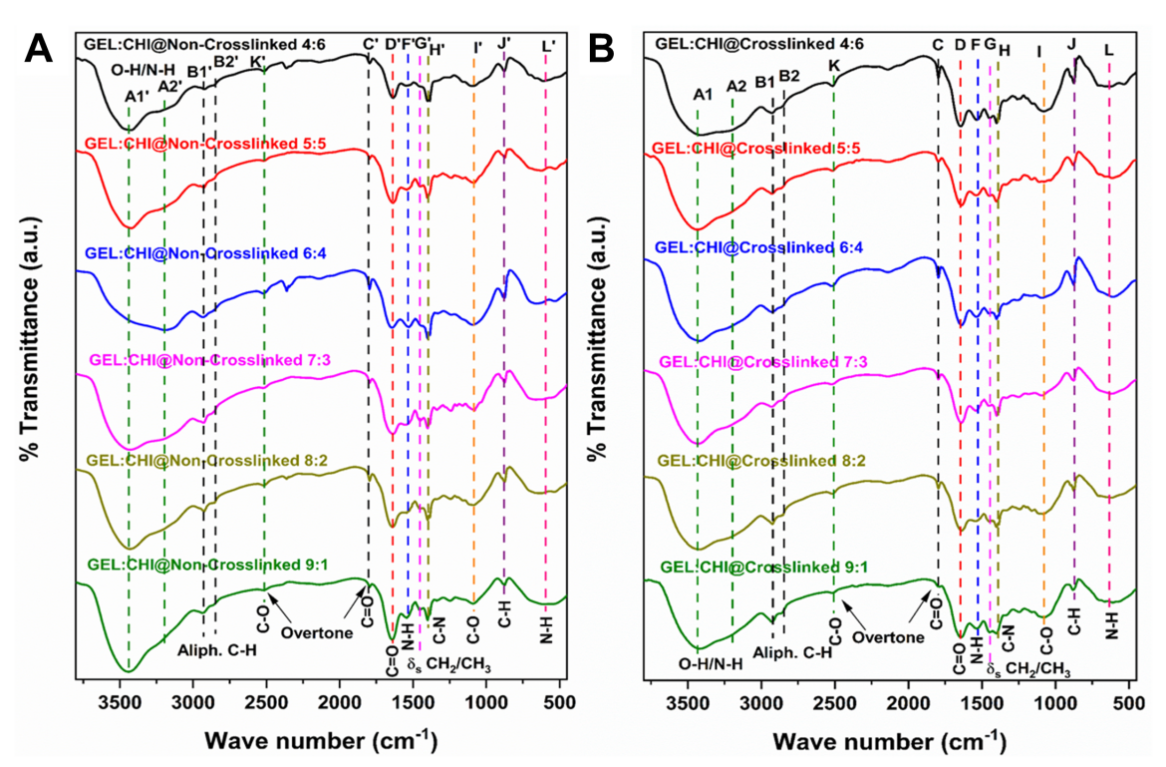


**Figure S3.** FTIR spectra of gelatin/chitosan NFM. FTIR spectra of non-crosslinked (A) and crosslinked (B) gelatin/chitosan NFM.


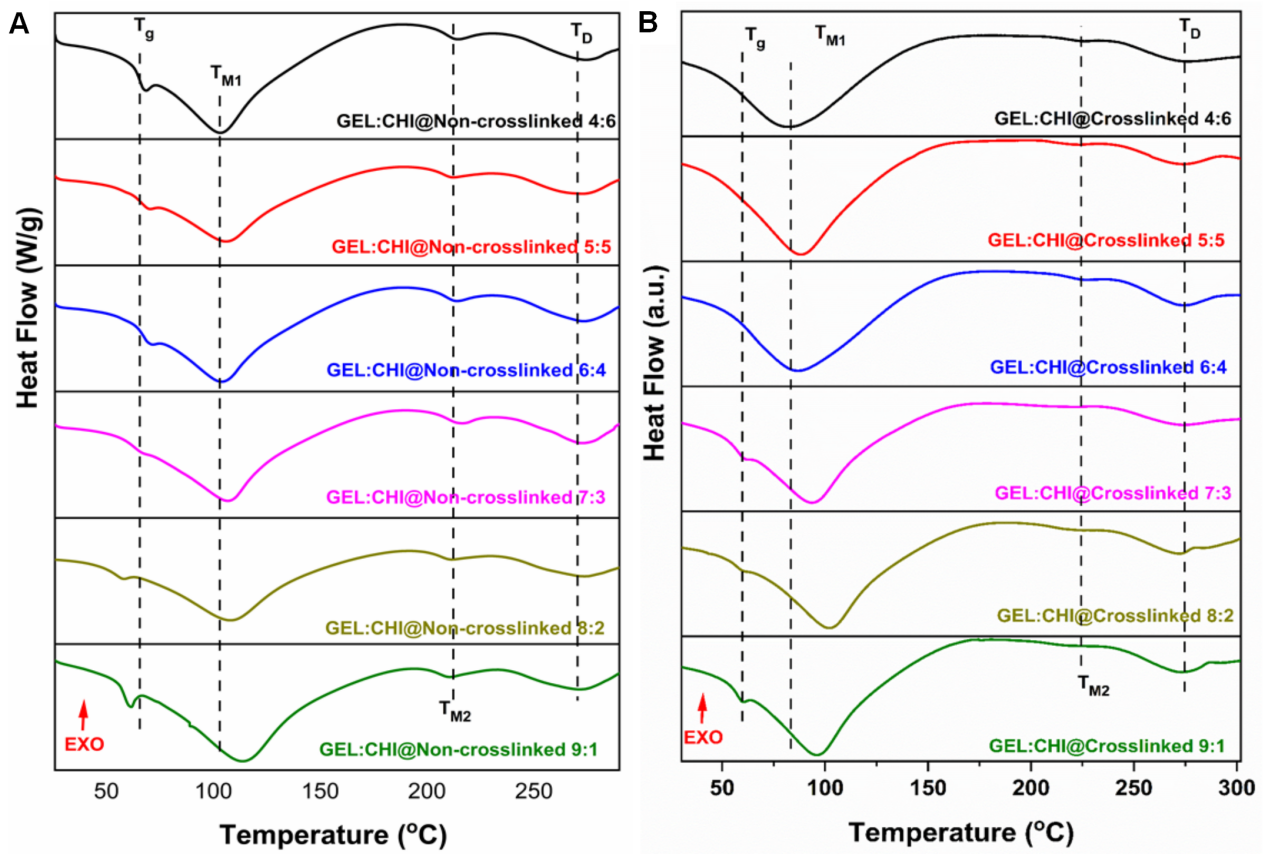


**Figure S4.** DSC thermograms of gelatin/chitosan NFM. DSC curves of non-crosslinked (A) and crosslinked (B) gelatin/chitosan NFM.


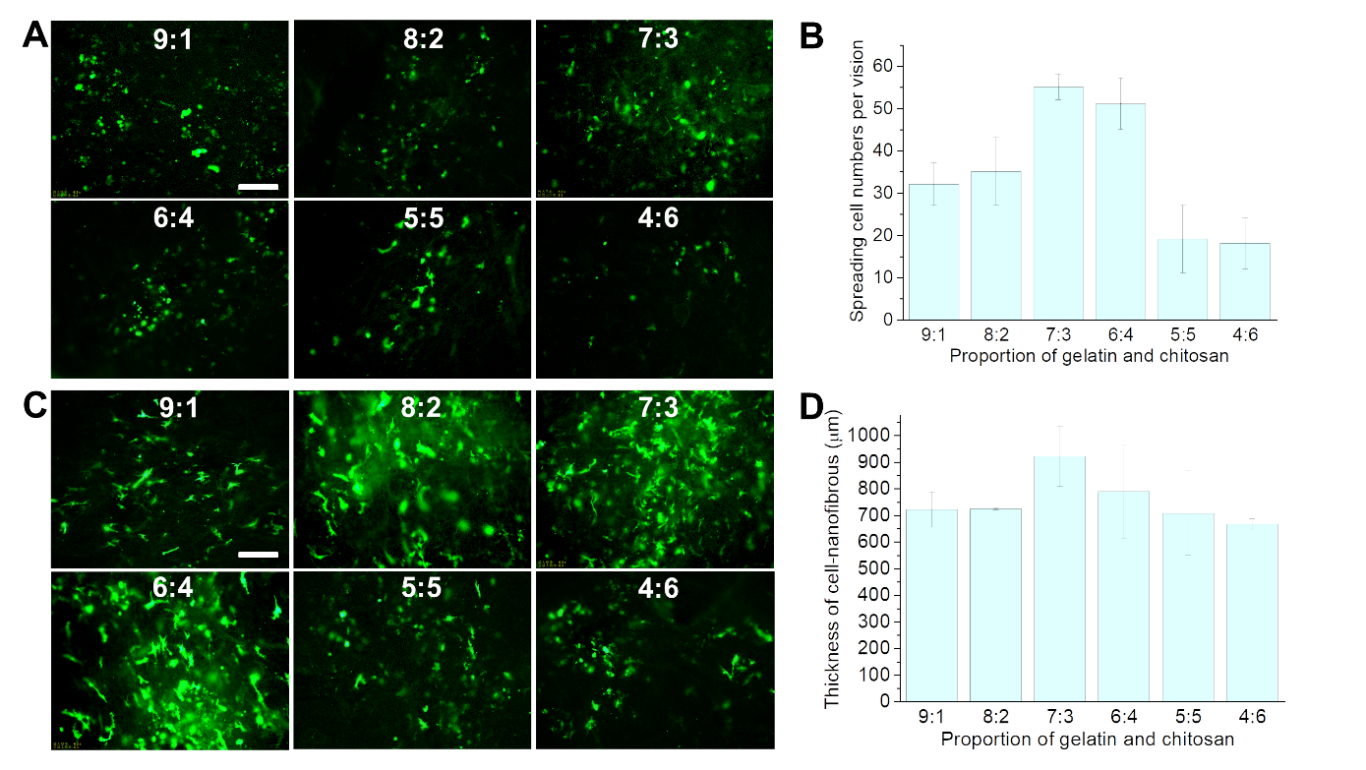


**Figure S5.** Adhesion and proliferation of ADSCs on NFM with different gelatin/chitosan proportions. (A) ADSC-GFP adhered on the NFM after one day cultivation. (B) The number of spreading cells on different gelatin/chitosan NFM. (C) After one week culture, ADSC-GFP almost coved the surface of NFM. Scale bars 200 μm. (D) The thickness of cell-NFM was measured by confocal laser microscope.


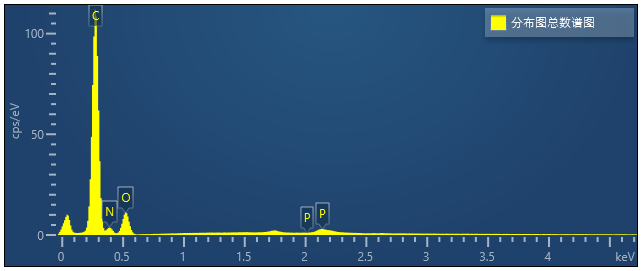


**Figure S6.** The composition of plasmid@NFM confirmed by energy dispersive X-ray spectroscopy.


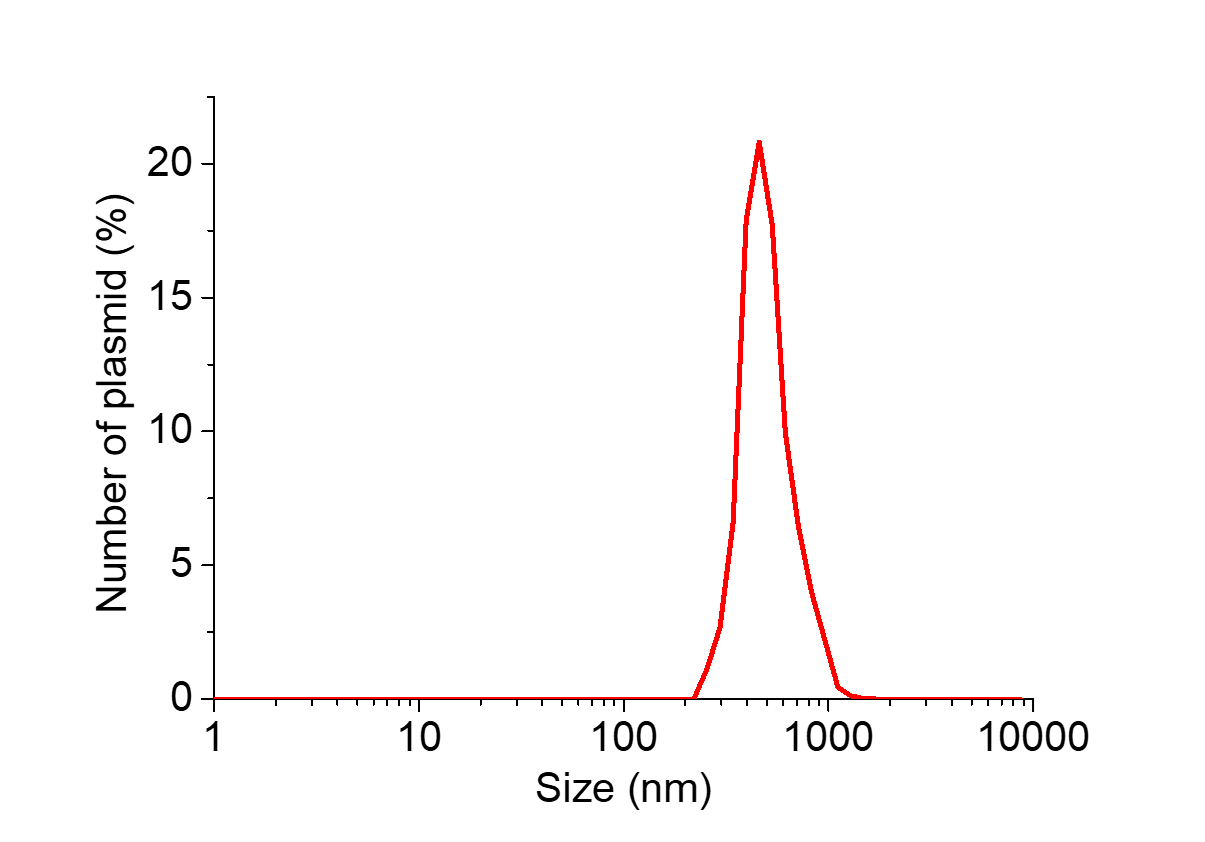


**Figure S7.** The size of VEGF plasmid measured by DLS.


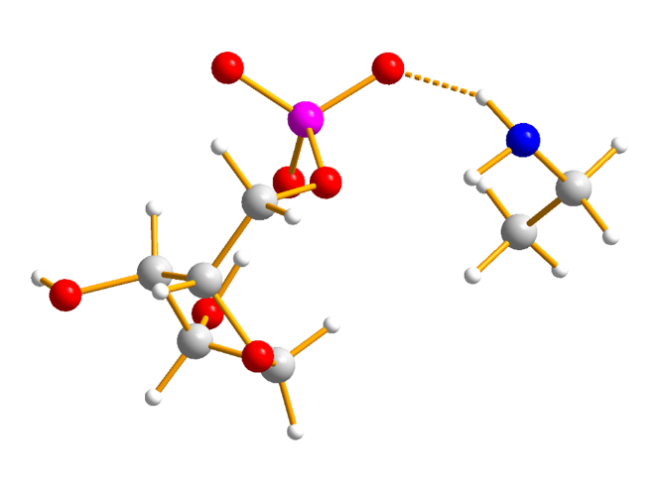


**Figure S8.** The interaction of gelatin/chitosan and plasmid.


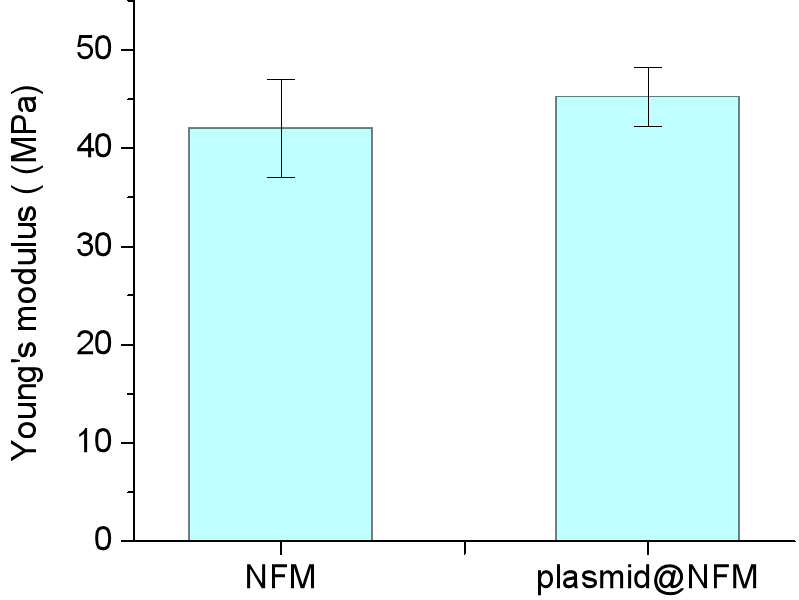


**Figure S9.** The mechanical property of plasmid@NFM.


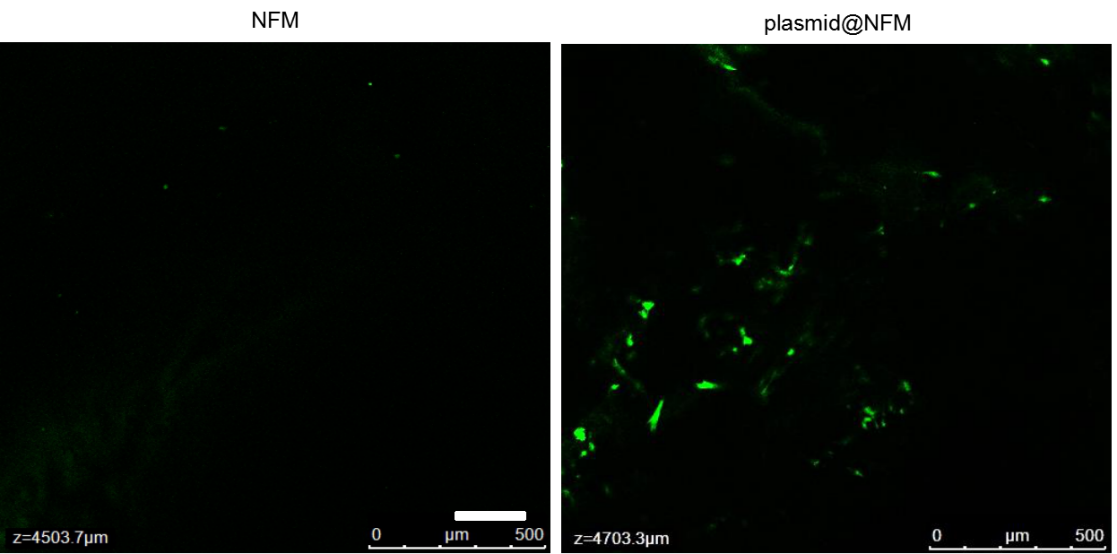


**Figure S10.** The expression of VEGF protein in ADSCs cultured on gelatin/chitosan (7:3) NFM or plasmid@NFM. Scale bar 200 μm.


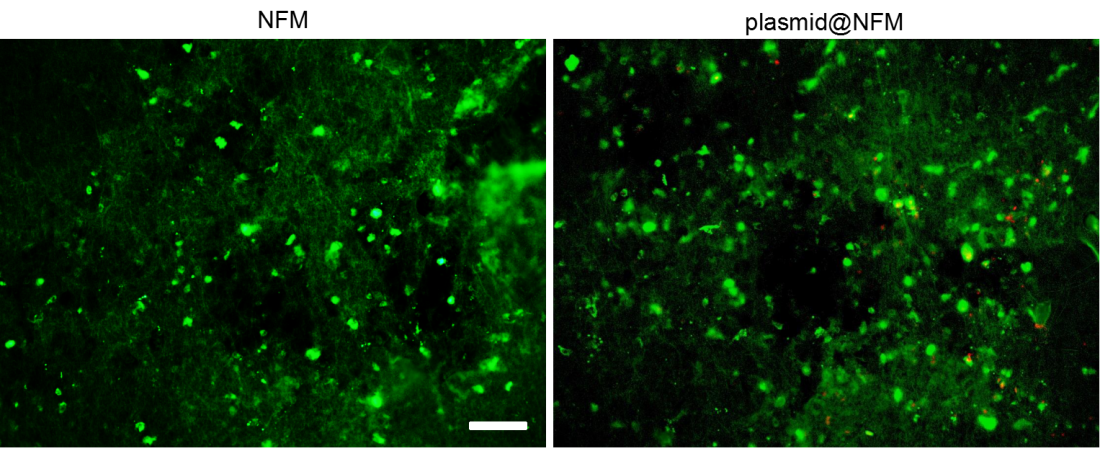


**Figure S11.** Live/dead cell staining after one-week culture on gelatin/chitosan (7:3) NFM or plasmid@NFM. Scale bar 200 μm.


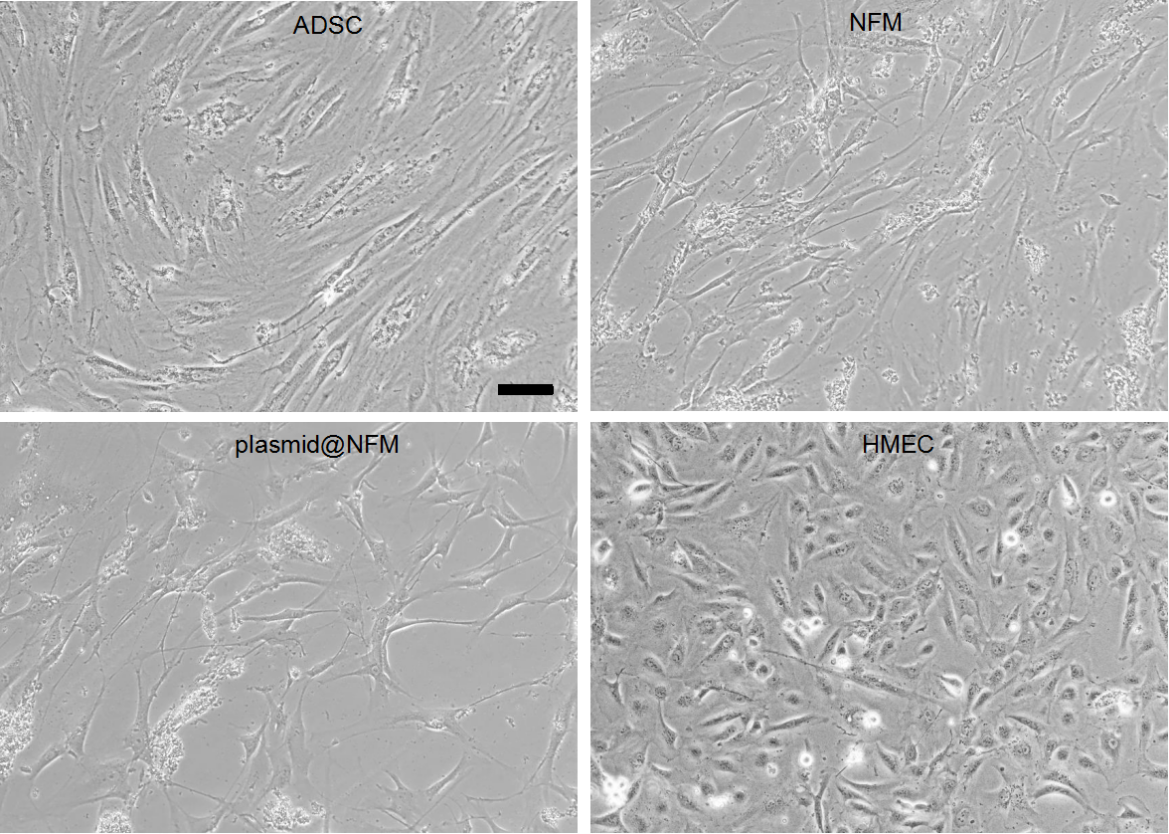


**Figure S12.** Morphological changes of ADSCs after culturing on NFM or plasmid@NFM for 3 weeks. Scale bar 100 μm. ADSC: ADSCs only (negative control). HMEC: human microvascular endothelial cell as positive control.


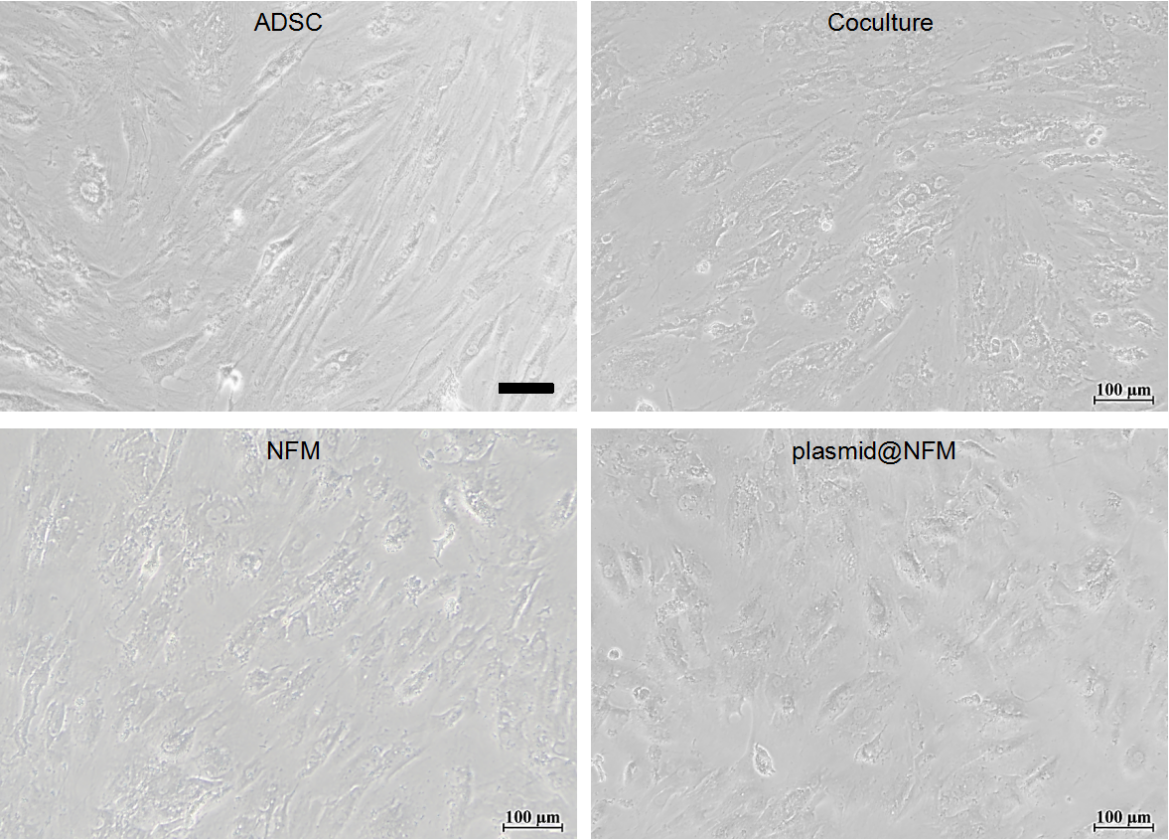


**Figure S13.** Morphological changes of ADSCs cultured on NFM or plasmid@NFM after coculturing with HaCat cells for 2 weeks. Scale bar 100 μm. ADSC: ADSC only as negative control; coculture: ADSC coculturing with HaCat by cell culture insert; NFM: ADSC cultured on NFM coculturing with HaCat by cell culture insert; plasmid-NFM: ADSC cultured on plasmid@NFM coculturing with HaCat by cell culture insert.


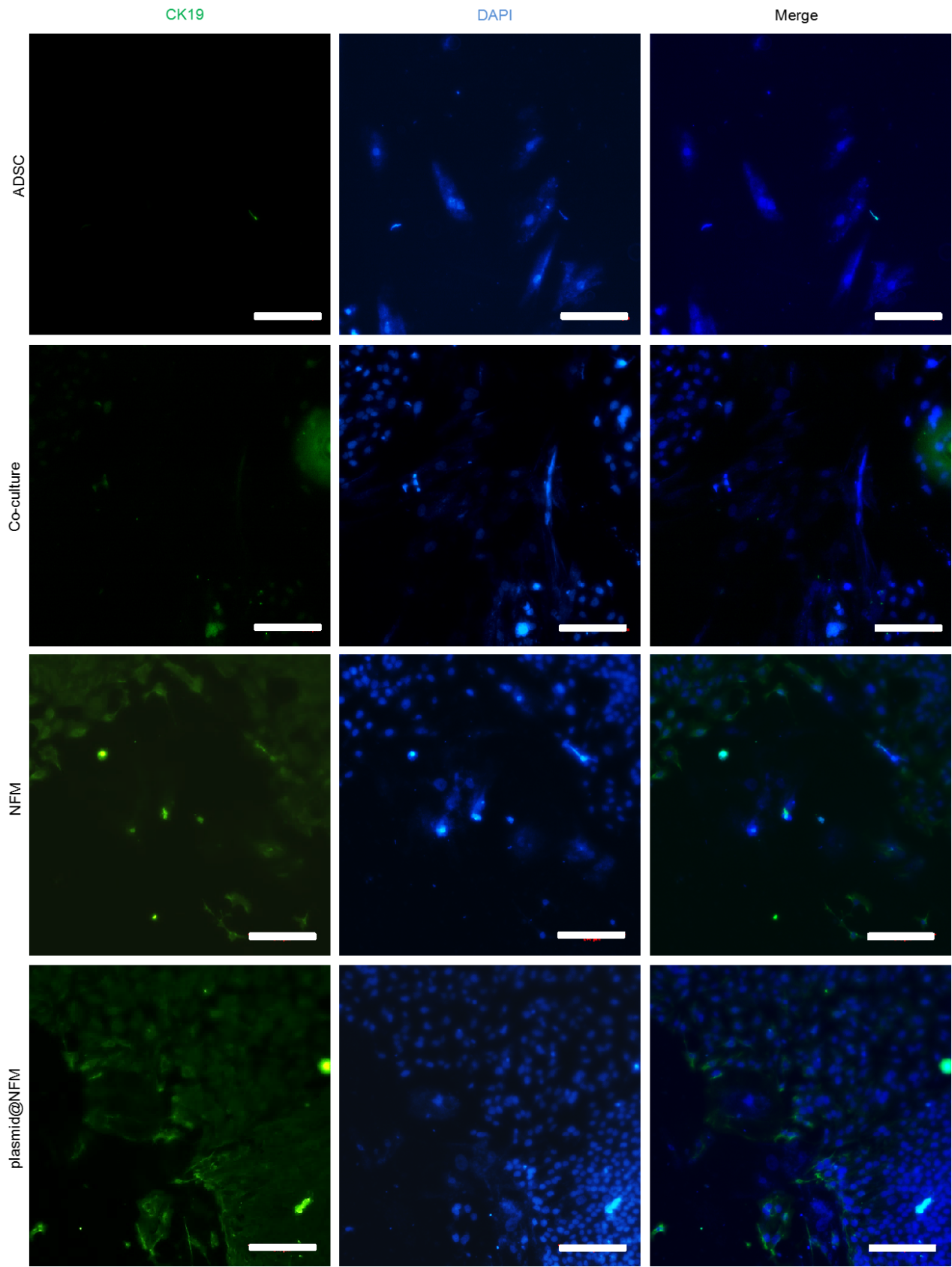


**Figure S14.** Protein expression of CK19 in differentiated ADSCs cultured on NFM or plasmid@NFM after coculturing with HaCat cells for 2 weeks. Scale bar 200 μm.


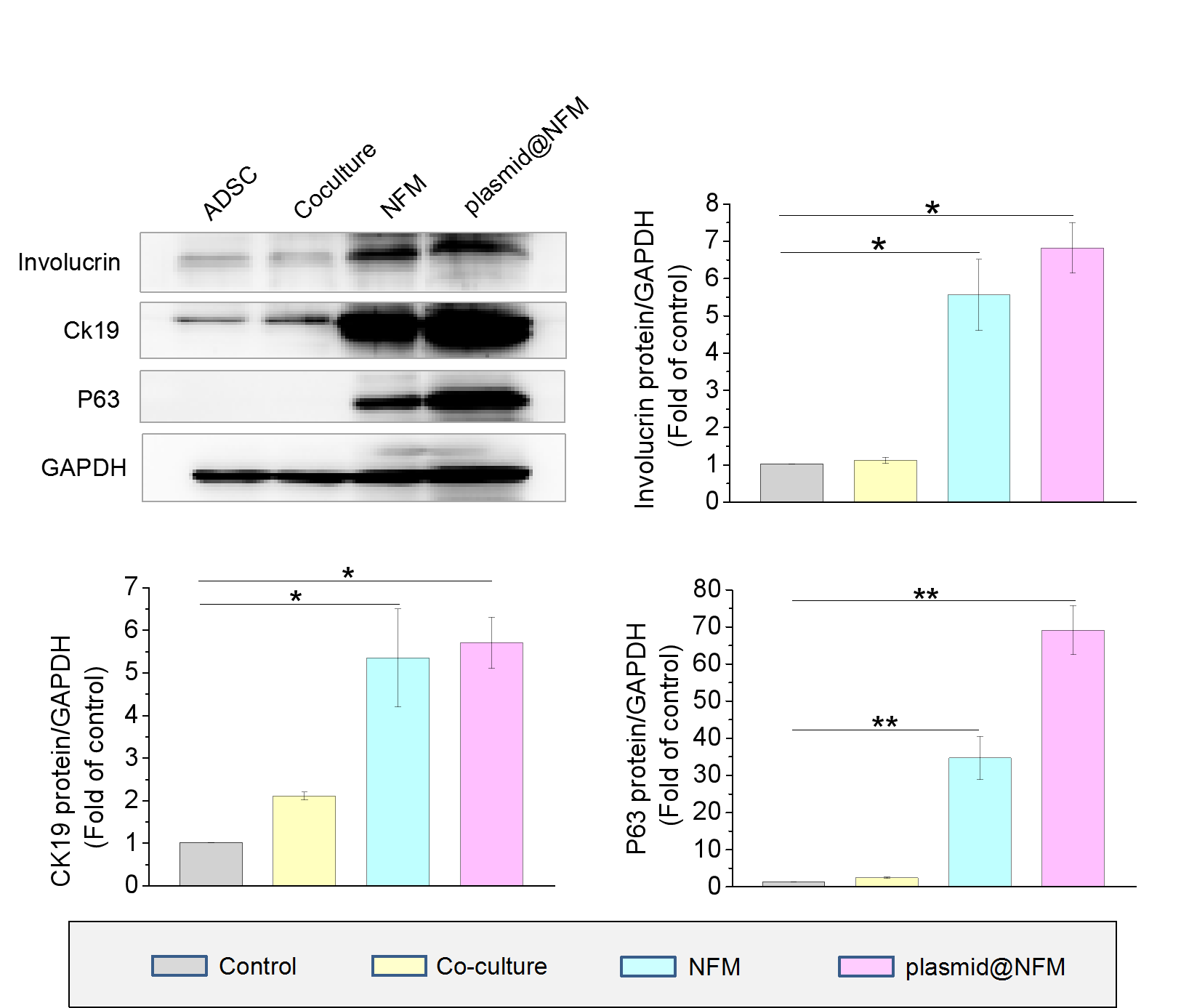


**Figure S15.** Keratinocyte-specific protein expression of differentiated ADSCs cultured on NFM or plasmid@NFM after coculturing with HaCat cells for 2 weeks. **p < 0.05* versus control group.


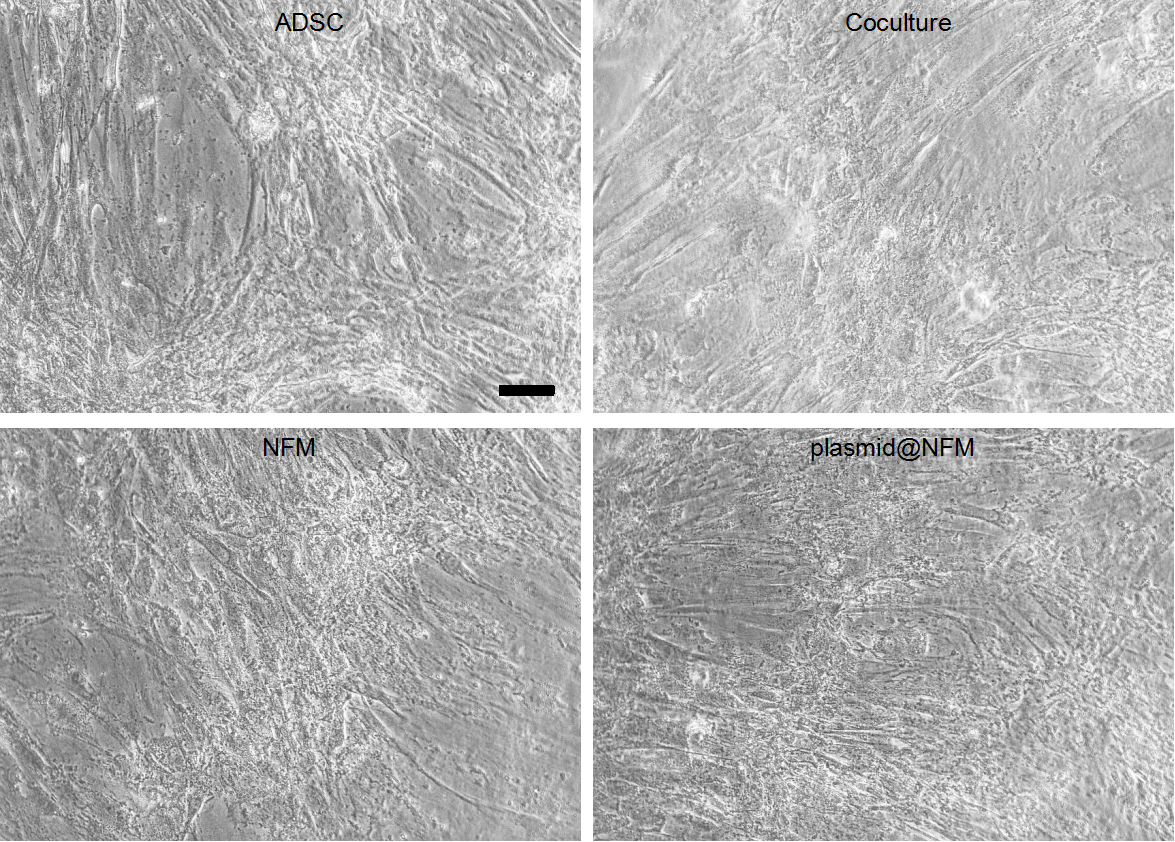


**Figure S16.** Morphological changes of ADSCs cultured on NFM or plasmid@NFM after coculturing with HSF cells for 2 weeks. Scale bar 100 μm.


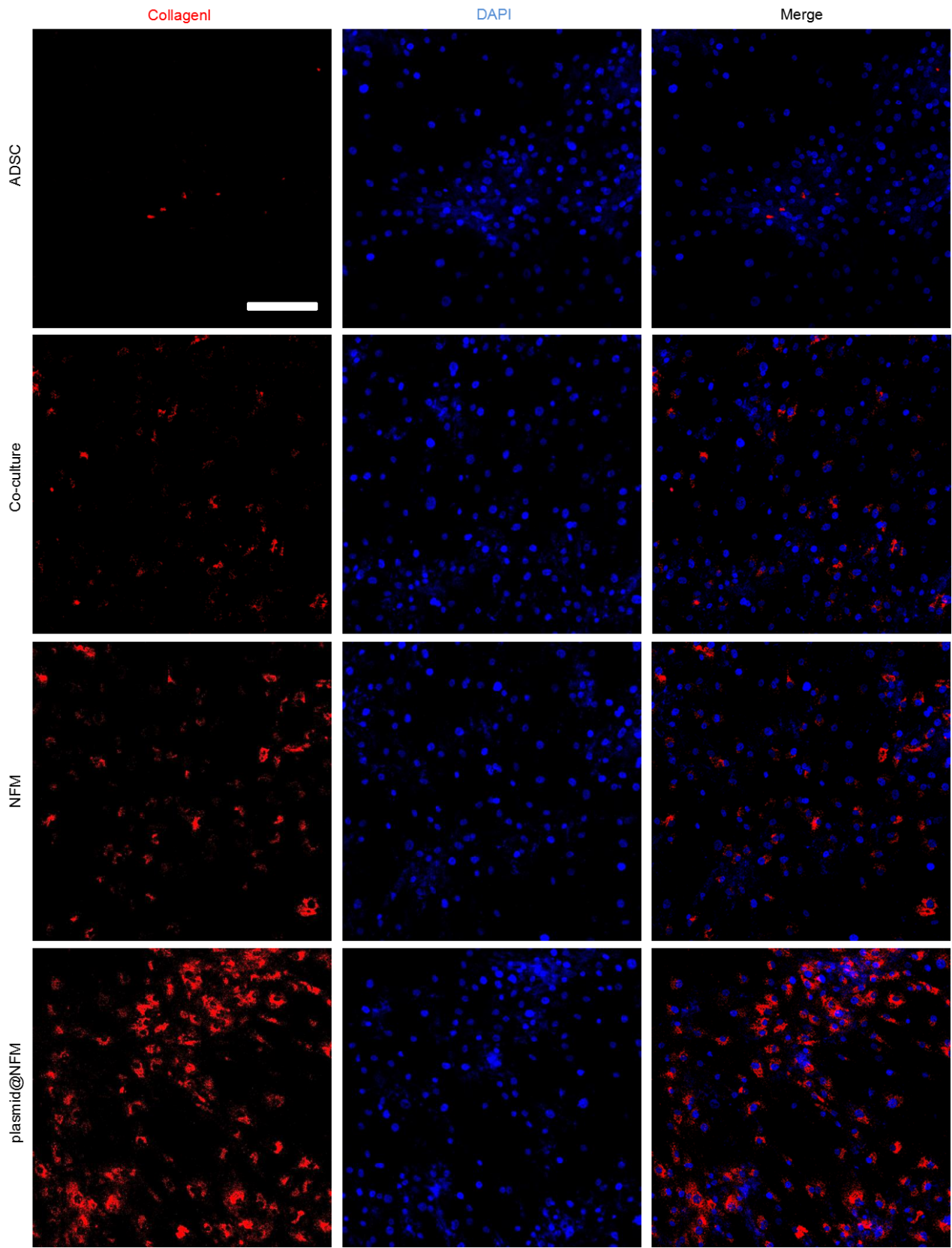


**Figure S17.** Protein expression of collagen I in differentiated ADSCs cultured on NFM or plasmid@NFM after coculturing with HSF cells for 2 weeks. Scale bar 200 μm.


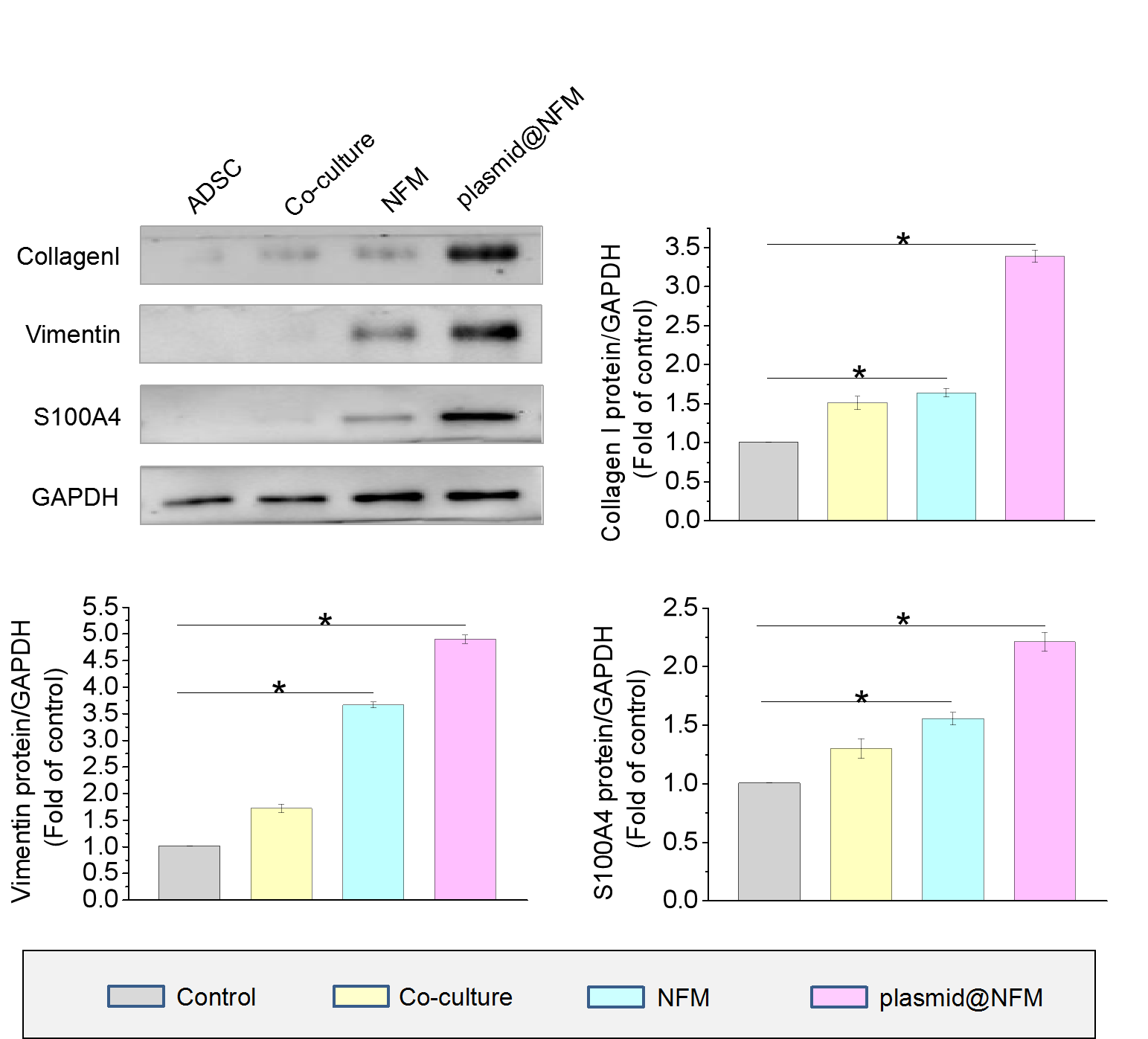


**Figure S18.** Fibroblast-specific protein expression of differentiated ADSCs cultured on NFM or plasmid@NFM after coculturing with HSF cells for 2 weeks. **p < 0.05*.


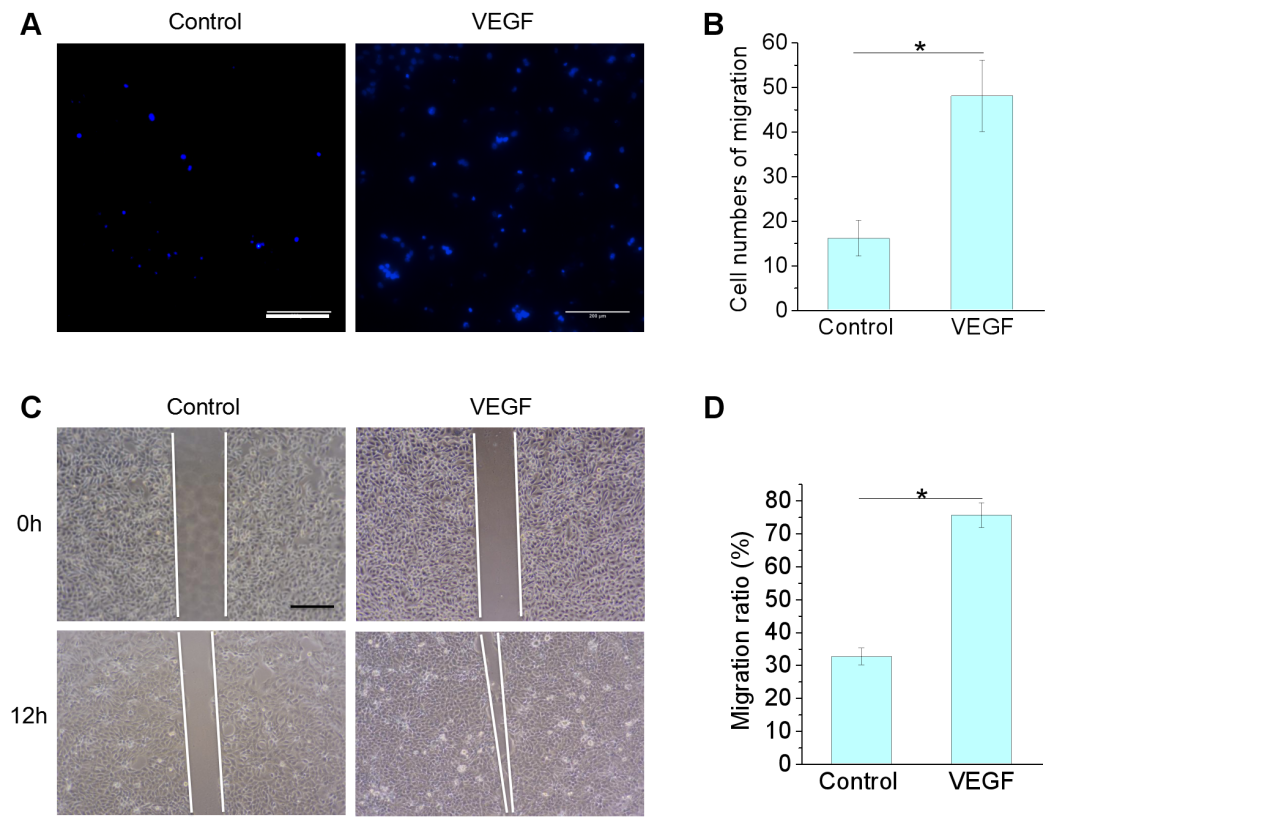


**Figure S19.** Migration of HaCat cells induced by VEGF. (A) The migration of HaCat cells induced by VEGF examined by transwell inserts, migrated HaCat cells showed by DAPI staining. Scale bar 200 μm. (B) Quantitative analysis of migrated cells. **p<0.05.* (C) The migration of HaCat cells tested by wound healing assay. Scale bar 200 μm. (D) Migration ratios of HaCat cells. **p<0.05.*


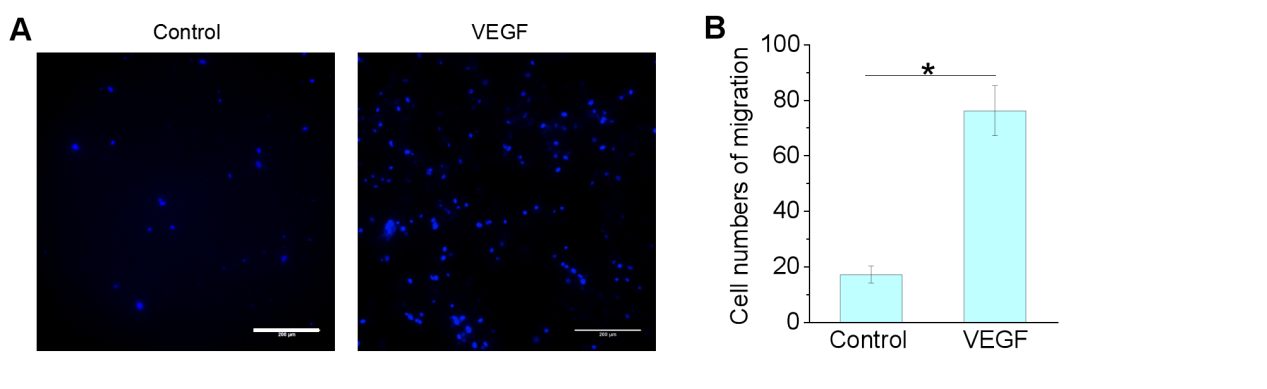


**Figure S20.** Migration of HSF cells induced by VEGF tested by transwell insert. (A) Migrated HSF cells showed by DAPI staining. Scale bar 200 μm. (B) Quantitative analysis of migrated cells. **p<0.05.*


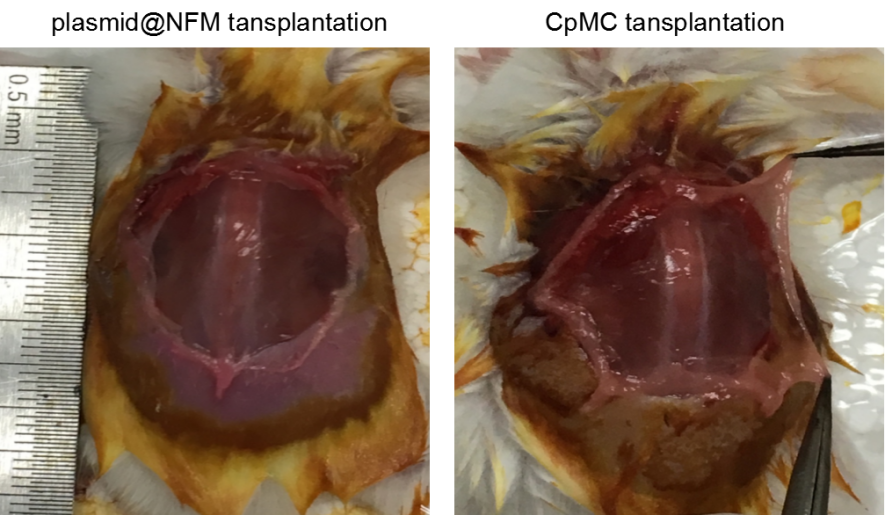


**Figure S21.** Transplantation of plasmid@NFM or CpMC onto the wound site.


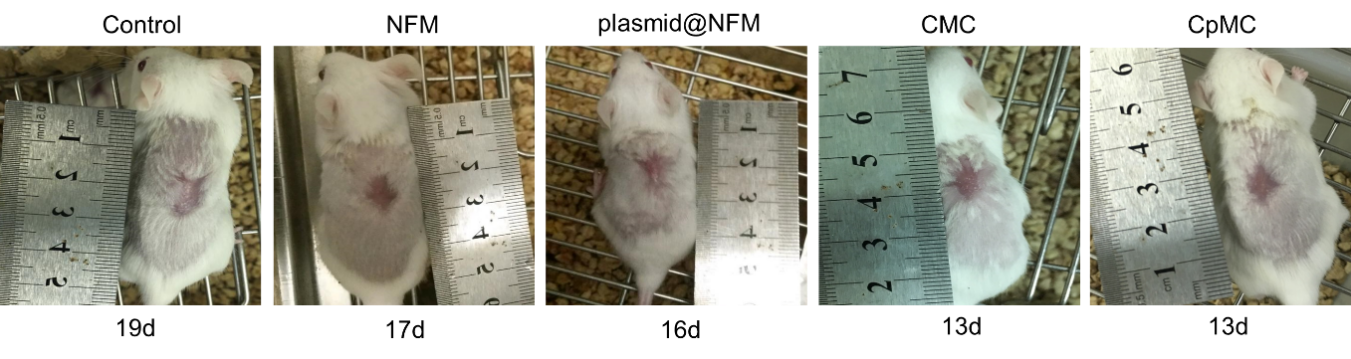


**Figure S22.** Wound with different treatment closed at different day.


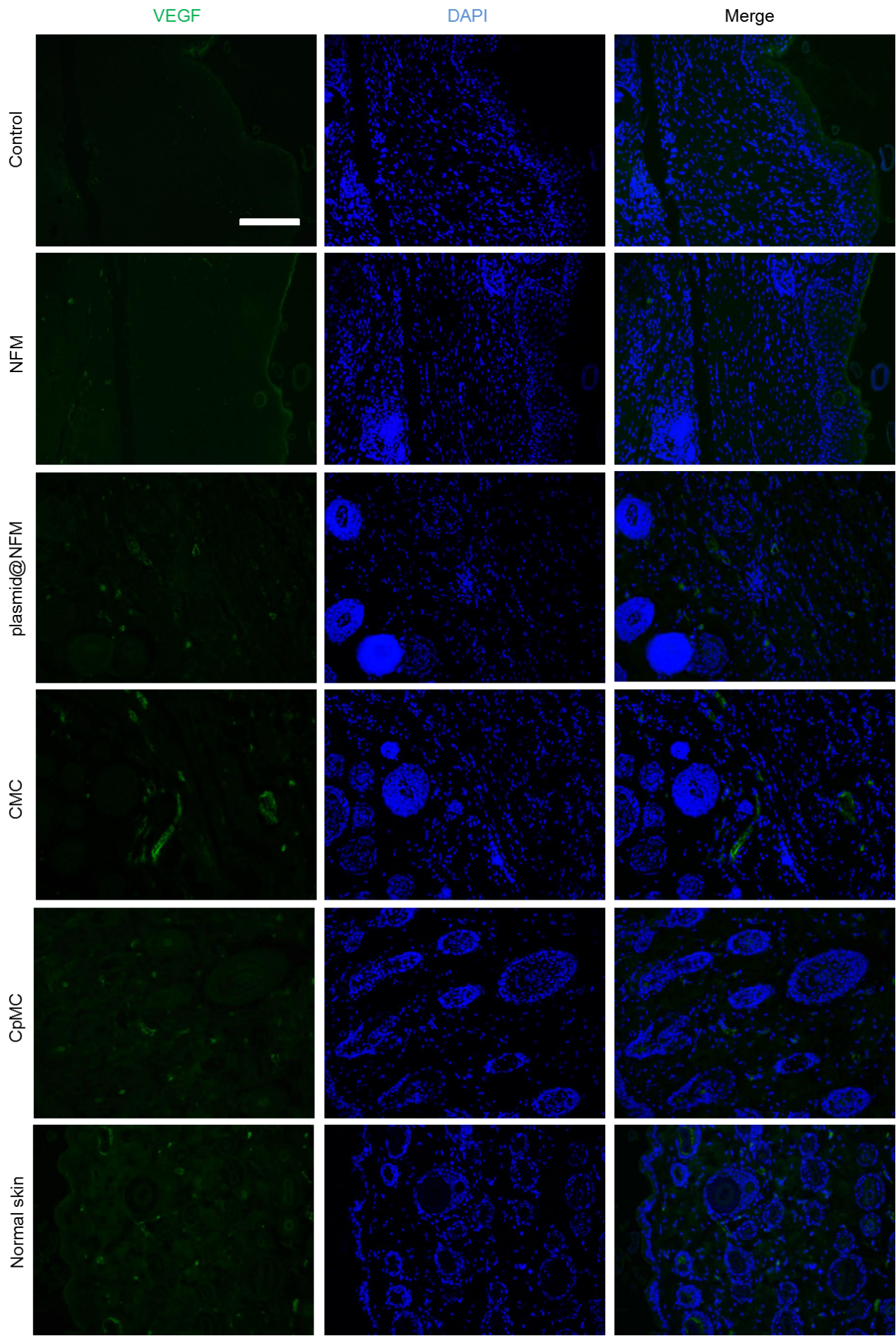


**Figure S23.** Immunofluorescent staining of VEGF (green) at the wound area after healing. Blue colour indicates the nuclei. Scale bar in all images were 100 μm.

**Table S1. Sequences of primers for RT-PCR.**

| **Gene** | **Forward** | **Reverse** |
| --- | --- | --- |
| vWF | CCCACCGGATGGCTAGGTATT | GAGGCGGATCTGTTTGAGGTT |
| CD31(PECAM) | GTCATGGCCATGGTCGAGTA | CTCCTCGGCGATCTTGCTGAA |
| VE-cadherin | ATTGAGACAGACCCCAAACG | TTCTGGTTTTCTGGCAGCTT |
| eNOS | GACCCTCACCGCTACAACAT | CTGGCCTTCTGCTCATTTTC |
| KDR(Flk-1) | TCTGTGGTTCTGCGTGGAGA | GTATCATTTCCAACCACCCT |
| GAPDH | TGCACCACCAACTGCTTAG | GATGCAGGGATGATGTTC |

**Table S2**. Characteristics of different Non crosslinked & crosslinked gelatin/chitosan NFM.

| **NFM (gelatin/chitosan)** | **Fiber diameter (μm)** | | **Pore size (μm)** | | **Young’s modulous (MPa)** | | **Swelling property**  **(%)** | **Residual mass after 1 month**  **degradation (%)** | **pH value** |
| --- | --- | --- | --- | --- | --- | --- | --- | --- | --- |
|  | **Non-crosslinked** | **Crosslinked** | **Non-crosslinked** | **Crosslinked** | **Non-crosslinked** | **Crosslinked** |  |  |  |
| **9:1** | 1.37±0.36 | 1.19±0.12 | 18.51±1.22 | 10.74±1.89 | 27.51±3.52 | 83.01±3.52 | 451.48±61.62 | 75.91±0.41 | 7.01±0.09 |
| **8:2** | 1.26±0.18 | 1.08±0.14 | 16.92±1.81 | 9.83±1.21 | 34.1±2.61 | 61.03±4.31 | 463.21±31.38 | 75.22±3.02 | 6.97±0.06 |
| **7:3** | 0.61±0.13 | 0.49±0.05 | 14.74±1.54 | 9.13±2.43 | 42.01±4.52 | 47.11±8.01 | 389.83±17.62 | 74.51±0.83 | 7.04±0.01 |
| **6:4** | 0.56±0.1 | 0.44±0.18 | 13±3.21 | 9.05±2.7 | 34.07±1.92 | 41.08±6.12 | 356.91±6.44 | 74.05±0.88 | 6.98±0.07 |
| **5:5** | 0.51±0.11 | 0.41±0.08 | 9.64±2.43 | 4.58±1.64 | 30.51±2.13 | 31.07±3.13 | 298.87±13.11 | 74.21±0.99 | 6.98±0.08 |
| **4:6** | 0.47±0.15 | 0.37±0.13 | 8.13±2.51 | 4.55±1.51 | 25.05±3.82 | 33.06±2.71 | 236.33±15.79 | 73.9±2.11 | 7.03±0.05 |

**Table S3.** DSC parameters of different Non crosslinked & crosslinked gelatin/chitosan NFM.

| **NFM (gelatin/chitosan)** | **Samples** | **T_g_ (^o^C)** | **T_m1_ (^o^C)** | **T_m2_ (^o^C)** | **T_d_ (^o^C)** | **ΔH(j/g)** |
| --- | --- | --- | --- | --- | --- | --- |
| **Non-Crosslinked** | 4:6 | 65 | 72-167 | 195-231 | 234-298 | 125.5 |
|  | 5:5 | 66 | 75-170 | 196-231 | 234-290 | 144.9 |
|  | 6:4 | 66 | 75-170 | 197-230 | 236-290 | 151.85 |
|  | 7:3 | 61 | 72-179 | 196-233 | 236-290 | 173.5 |
|  | 8:2 | 54 | 65-183 | 195-232 | 232-295 | 259.44 |
|  | 9:1 | 58 | 66-183 | 195-231 | 233-291 | 320.69 |
| **Crosslinked** | 4:6 | - | 38-159 | 213-237 | 241-298 |  |
|  | 5:5 | - | 40-158 | 207-230 | 233-292 | - |
|  | 6:4 | 56 | 58-163 | 211-238 | 243-291 | 131.53 |
|  | 7:3 | 57 | 65-162 | 200-230 | 237-295 | 126.25 |
|  | 8:2 | 56.33 | 63-178 | 204-235 | 239-279 | 205.67 |
|  | 9:1 | 56.6 | 63-170 | 201-235 | 236-285 | 151.67 |
